# *ACTN3* genotype influences androgen response in developing murine skeletal muscle

**DOI:** 10.1126/sciadv.adw1059

**Published:** 2025-08-27

**Authors:** Kelly N. Roeszler, Michael See, Lyra R. Meehan, Giscard Lima, Alexander Kolliari-Turner, Sarah E. Alexander, Shanie Landen, Harrison D. Wood, Chrystal F. Tiong, Weiyi Chen, Tomris Mustafa, Peter J. Houweling, Nir Eynon, Severine Lamon, Yannis Pitsiladis, David J. Handelsman, Fernando J. Rossello, Mirana Ramialison, Kathryn N. North, Jane T. Seto

**Affiliations:** ^1^ Murdoch Children’s Research Institute, The Royal Children’s Hospital, Melbourne, Victoria, Australia.; ^2^ Department of Paediatrics, University of Melbourne, The Royal Children’s Hospital, Melbourne, Victoria, Australia.; ^3^ ANZAC Research Institute, University of Sydney and Andrology Department, Concord Hospital.; ^4^ School of Sport and Health Sciences, University of Brighton, Eastbourne, UK.; ^5^ Cardiometabolic Health and Exercise Physiology, Baker Heart and Diabetes Institute, Melbourne, Australia.; ^6^ Centre for Endocrinology and Metabolism, Hudson Institute of Medical Research, Melbourne, Victoria, Australia.; ^7^ Department of Physiology, Monash University, Melbourne, Victoria, Australia.; ^8^ Neuronal Control of Metabolism, Max Planck Institute for Metabolism Research, Cologne, North Rhine-Westphalia, Germany.; ^9^ Australian Regenerative Medicine Institute (ARMI), Faculty of Medicine, Nursing and Health Sciences, Monash University, Melbourne, Victoria, Australia.; ^10^ School of Exercise and Nutrition Sciences, Faculty of Health, Deakin University, Burwood, Australia.; ^11^ Department of Sport, Physical Education and Health, Hong Kong Baptist University, Kowloon Tong, Hong Kong.; ^12^ Novo Nordisk Foundation Center for Stem Cell Medicine, Murdoch Children’s Research Institute, Melbourne, Victoria 3052, Australia.; ^13^ Department of Clinical Pathology, University of Melbourne, Melbourne, Victoria, Australia.; ^14^ Australian Regenerative Medicine Institute (ARMI), Monash University, Melbourne, Victoria, Australia.

## Abstract

Androgens act through androgen receptor (AR) to maintain muscle mass. Evidence suggests that this pathway is influenced by “the gene for speed,” *ACTN3* (α-actinin-3). Given that one in five people lack α-actinin-3, it is possible that they may respond to androgens differently. Here, we show that α-actinin-3 deficiency decreases AR in muscles of mice and humans (in males and females) and that AR positively correlates with α-actinin-3 expression in a dosage-dependent manner. α-Actinin-3 deficiency exacerbates gastrocnemius mass loss with androgen deprivation in male mice and stunts the muscle growth response to dihydrotestosterone in female mice at the onset of puberty. This is mediated by differential activation of pathways regulating amino acid metabolism, intracellular transport, autophagy, mitochondrial activity, MAPK, and calcineurin signaling, likely driven by seven key genes that are both androgen sensitive and α-actinin-3–dependent in expression. Our results highlight a role for *ACTN3* as a regulator of muscle mass and a genetic modifier of androgen action in skeletal muscle.

## Teaser

ACTN3 deficiency (which is common in humans) reduces muscle androgen receptor levels and modifies muscle response to testosterone.

## INTRODUCTION

Androgens orchestrate the development and maintenance of masculine reproductive characteristics and have well-characterized anabolic actions in skeletal muscle, heart, and bone (*1*). Testosterone and its more potent metabolite dihydrotestosterone (DHT) are the primary androgens in male mammals and are present at relatively lower levels in females (*2*). Testosterone decline [due to hypogonadism, aging, or androgen deprivation therapy (ADT) to treat prostate cancer] is associated with muscle wasting (*3*, *4*), while testosterone replacement therapy is prescribed at physiological doses to men whose endogenous testosterone production is severely limited to maintain or restore muscle mass (*1*).

The anabolic actions of androgens in muscles are, in part, exerted by binding to the androgen receptor (AR) as a ligand-activated transcription factor activating androgen action. Androgen action involves stimulation of protein synthesis pathways (such as mTORC1 and calcineurin signaling) and inhibition of protein degradation (via suppressing E3-ubiquitin ligase genes *Fbxo32* and *Trim63*) (*4*, *5*). Androgens also directly promote expression of target genes such as myostatin (*Mstn*), a key repressor of muscle growth as a negative feedback mechanism to restrain unlimited muscle growth (*6*), as well as genes involved in induction of autophagy and myoblast proliferation (*7*–*9*). In addition, activation of AR involves recruitment of coactivators such as α-actinin-2 and glucocorticoid receptor interacting protein 1 to facilitate transcription of target genes (*10*).

α-Actinin-2 (*ACTN2*) and α-actinin-3 (*ACTN3*) are major components of the muscle contractile apparatus. α-Actinin-2 is present in all muscle fibers, while α-actinin-3 has developed specialized expression in only type 2 (fast-twitch and glycolytic) fibers, which are important for rapid, repetitive muscle contractions and for activities that require speed and power (*11*). A common null polymorphism in *ACTN3* (R577X) arose in modern humans and underwent positive selection ~10 to 15,000 years ago (*12*, *13*). One in five people worldwide are completely α-actinin-3 deficient due to inheriting two copies of the null variant (577X) in the *ACTN3* gene (*14*). α-Actinin-3 deficiency does not cause disease because of compensatory up-regulation of α-actinin-2, which shares high sequence similarity (*11*). However, the absence of α-actinin-3 is detrimental to sprint and power performance in elite athletes (hence, became known as “the gene for speed”) and the general population (*15*–*18*). Among the elderly, α-actinin-3 deficiency is associated with significantly lower strength and higher frailty scores in an elderly Chinese population (*19*), and a meta-analysis using two large independent cohorts of Caucasian postmenopausal women showed that carriage of the *ACTN3* 577X allele increases the risk of falling by 33% (*20*).

Accumulating evidence in humans and mouse models suggests a relationship between α-actinin-3 expression and androgen response. In a cohort of elite male and female Russian athletes, carriage of the *ACTN3* 577R allele was associated with significantly higher testosterone levels compared to XX individuals (*21*). Testicular feminized mice (*Tfm*; which have an inactivating mutation in AR) and hypogonadal mice (*hpg*; which lack gonadotropin and sex steroid production) are both androgen deficient and both show significantly reduced *Actn3* expression in the testis, while testosterone treatment of *hpg* mice up-regulated *Actn3*, confirming that *Actn3* is an androgen-regulated target gene in the testis (*22*). Comparison of AR knockout (ARKO) mice with the *Actn3* KO mouse model also revealed many similarities in muscle characteristics. Relative to wild-type (WT) mice, both male ARKO and *Actn3* KO mice demonstrate decreased body weight, reductions in hind-limb muscle mass and strength, increased calcineurin signaling, increased expression of slow-twitch contractile proteins, and enhanced fatigue resistance (*23*–*25*). Similarly, both male *Actn3* KO and the fast-twitch muscle-specific ARKO show reduced bone mineral density (BMD) and reduced baseline expression of *Mstn*, *Fbxo32*, and *Trim63*, critical genes that regulate and maintain muscle mass (*26*–*28*). These results suggest that changes in androgen response with *ACTN3* genotype underlie the alterations in muscle performance associated with α-actinin-3 deficiency.

On this basis, we hypothesized that *ACTN3* genotype would alter baseline AR expression and, consequently, the muscle wasting response to androgen deprivation and muscle growth response to androgen treatments. We have previously shown that α-actinin-3 deficiency changes how muscles adapt following denervation and immobilization and protects against dexamethasone-induced muscle wasting through increases in protein synthesis pathways mTORC1 and calcineurin signaling and suppressing protein degradation (*27*, *29*). In this study, we examined the effect of α-actinin-3 deficiency on AR signaling and the skeletal muscle response to changes in circulating androgens.

## RESULTS

### AR protein expression is reduced in α-actinin-3–deficient human skeletal muscles

**Fig. 1.**
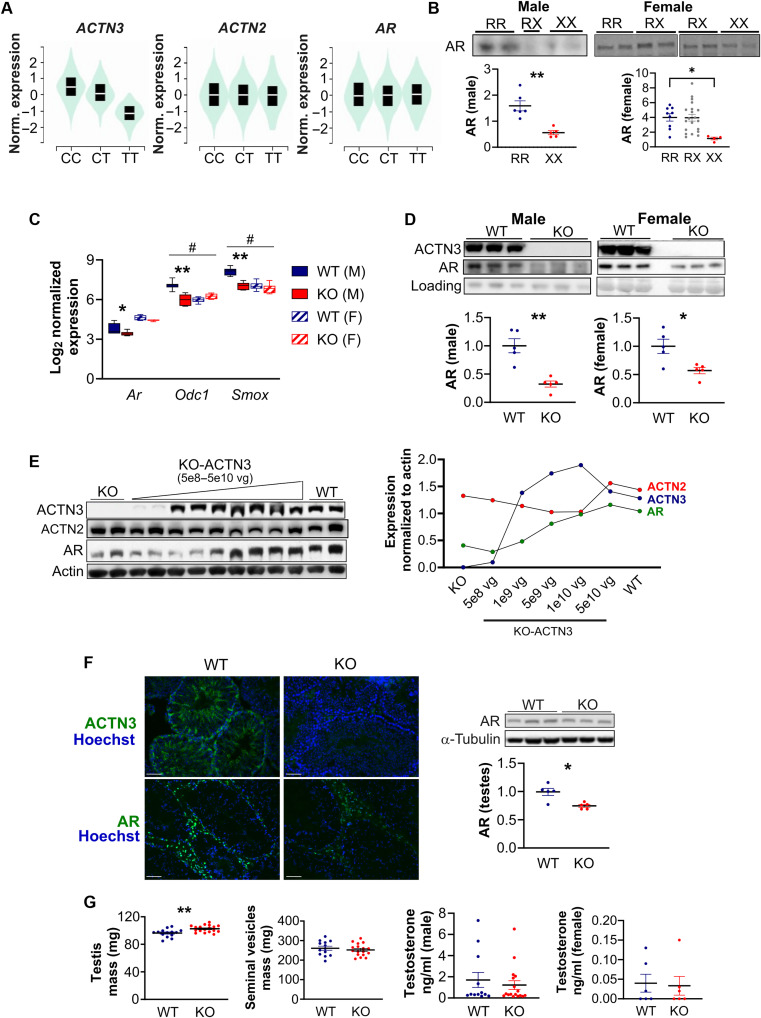
AR protein expression is reduced with α-actinin-3 deficiency in skeletal muscle and testis. (**A**) Trans-eQTL scan in the GTEx skeletal muscle dataset show that the R577X variant is significantly associated with *ACTN3* gene expression, but not for *ACTN2* or *AR.* (**B**) Protein expression of AR is significantly reduced in muscles from 577XX individuals compared to those from 577RR in males and females. (**C**) Expression of *Ar* and *Ar-*responsive genes (*Odc1* and *Smox*) is lower in *Actn3* KO muscles relative to that in WT in male, but not female, mice. (**D**) AR protein expression is lower in gastrocnemius muscles from male and female *Actn3* KO mice compared to those from WT. (**E**) Delivery of rAAV*-*CMV-*ACTN3* (5 × 10^8^ to 5 × 10^10^ vg) in *Actn3* KO muscles increased α-actinin-3 expression up to 1 × 10^10^ vg and decreased α-actinin-2, while AR expression is positively correlated with vector dosage. (**F**) Immunohistochemistry show absence of ACTN3 in KO testes and reduced AR staining compared to that in WT; reduced AR expression in KO testis is quantified by Western blot. (**G**) Testis mass is greater in KO mice compared to that in WT, but seminal vesicle mass and serum testosterone, as measured by radioimmunoassay assay (males) and mass spectrometry (females), are not different between *Actn3* genotypes. Data are represented as means ± SEM. *P < 0.05 and **P < 0.01 by Mann-Whitney *U* test; #*P* < 0.05 by two-way analysis of variance (ANOVA).

We performed a trans-expression quantitative trait locus (trans-eQTL) scan in the GTEx skeletal muscle dataset [GTEx Analysis Release V8 (dbGaP Accession phs000424.v8.p2)] to determine whether the *ACTN3* R577X variant (rs1815739) is associated with the expression of *ACTN3*, *ACTN2*, and *AR* (Fig. 1A). Consistent with our previous analysis using an earlier GTEx release (*30*), the R577X variant is significantly associated with *ACTN3* gene expression in skeletal muscle (*P* = 7.1 × 10^−141^), but not for *ACTN2.* Normalized gene expression of *AR* is also not significantly different between *ACTN3* R577X genotypes (Fig. 1A). However, quantitative polymerase chain reaction (qPCR) analysis of *AR* in vastus lateralis muscles from a small cohort of moderately trained Caucasian men (aged 18 to 47; *n* = 24) and women (aged 21 to 45; *n* = 20) show a trend for reduced *AR* expression in 577XX individuals compared to that in RR + RX (fig. S1A). We then examined protein expression of AR in human skeletal muscles from a cohort of young Caucasian men (aged 22 to 42, *n* = 12) and women (aged 18 to 37, *n* = 24) to determine whether AR levels are altered in association with *ACTN3* R577X genotype. Our results show that muscle AR is significantly reduced in XX individuals compared to that in RR in both male and female cohorts by 65% (*P* = 0.0043) and 72% (*P* = 0.0112), respectively (Fig. 1B).

### AR is reduced in *Actn3* knockout mouse muscles and increases with ACTN3 expression

We further examined the expression of *Ar* and *Ar-*responsive polyamine biosynthesis genes (*Odc1* and *Smox*) (*7*) in male and female WT and *Actn3* KO mouse muscles at baseline. Expression of these genes is lower in *Actn3* KO muscles compared to that in WT in male, but not female, mice (Fig. 1C); two-way analysis of variance (ANOVA) showed significant main and interaction effects between *Actn3* genotype and sex for *Odc1* (*F*_1,21_ = 31.76, *P* < 0.0001) and *Smox* (*F*_1,21_ = 12.55, *P* = 0.0019). At the protein level, *Actn3* KO gastrocnemius muscles showed 67.6 and 43% lower levels of AR in male (*P* = 0.0079) and female mice (*P* = 0.0317), respectively, compared to those in WT (Fig. 1D). Consistent with reductions in total AR expression with α-actinin-3 deficiency, phosphorylation of AR at Ser^213/210^ and Ser^791/790^, which marks AR for degradation (*31*), is higher in *Actn3* KO muscles by 59.1% (fig. S1B).

Estrogen receptor (ER) and thyroid receptor (TR) signaling were also examined, given their involvement in skeletal muscle myogenesis, metabolism, and contraction (*32*), and α-actinin-2 and α-actinin-4 have been shown to directly interact with these nuclear receptors and potentiate their activation (*10*, *33*). In contrast to AR, expression of genes associated with ER (*Esr1*, *Esrra*, *Esrrb*, and *Esrrg*) and TR (*Thra*, *Thrb*, and *Thrsp*) is similar between WT and *Actn3* KO muscles, regardless of sex (table S1). ER-α and ER-β protein expression is also similar in WT and *Actn3* KO muscles (fig. S1, C and D).

To determine whether AR protein expression is associated with α-actinin-3 dosage, variable doses of recombinant adeno-associated virus (rAAV)*–*cytomegalovirus promoter (CMV)–*ACTN3* [5 × 10^8^ to 5 × 10^10^ vector genomes (vg)] were delivered by intramuscular injection into the tibialis anterior (TA) muscles of *Actn3* KO mice, and AR expression was assessed (Fig. 1E). Results show a rise in α-actinin-3 expression in KO muscles with escalating doses of rAAV-CMV-*ACTN3* up to 1 × 10^10^ vg, dropping at 5 × 10^10^ vg. Consistent with the regulation of total sarcomeric α-actinin content (*18*), a reciprocal decrease in α-actinin-2 expression is also observed up to 1 × 10^10^ vg, which then increases at 5 × 10^10^ vg. In contrast, AR expression is positively correlated with rAAV-CMV-*ACTN3* dosage.

### AR is reduced in *Actn3* knockout mouse testes

We further examined the effects of α-actinin-3 deficiency on AR expression in the mouse testis because GTEX analysis indicates that *ACTN3* R577X also influences *ACTN3* expression in human testis (fig. S1E). Immunostaining in WT testis shows α-actinin-3 expression within the seminiferous tubules, while AR labeling is strongest in the interstitial compartment that coincide with the location of Leydig cells (*34*). In contrast, α-actinin-3 is absent in the testis of *Actn3* KO mice, while AR immunolabeling is reduced (Fig. 1F). Furthermore, testis mass is 6.5% higher in *Actn3* KO mice compared to that in WT (*P* = 0.0032), but seminal vesicle mass is not different between WT and *Actn3* KO mice (Fig. 1G). Despite changes in AR expression in the testis with α-actinin-3 deficiency, radioimmunoassay assay and mass spectrometry show similar levels of serum testosterone and luteinizing hormone between *Actn3* genotypes in male and female mice (Fig. 1G and fig. S1F).

### α-Actinin-3 deficiency increases muscle wasting induced by androgen deprivation

**Fig. 2.**
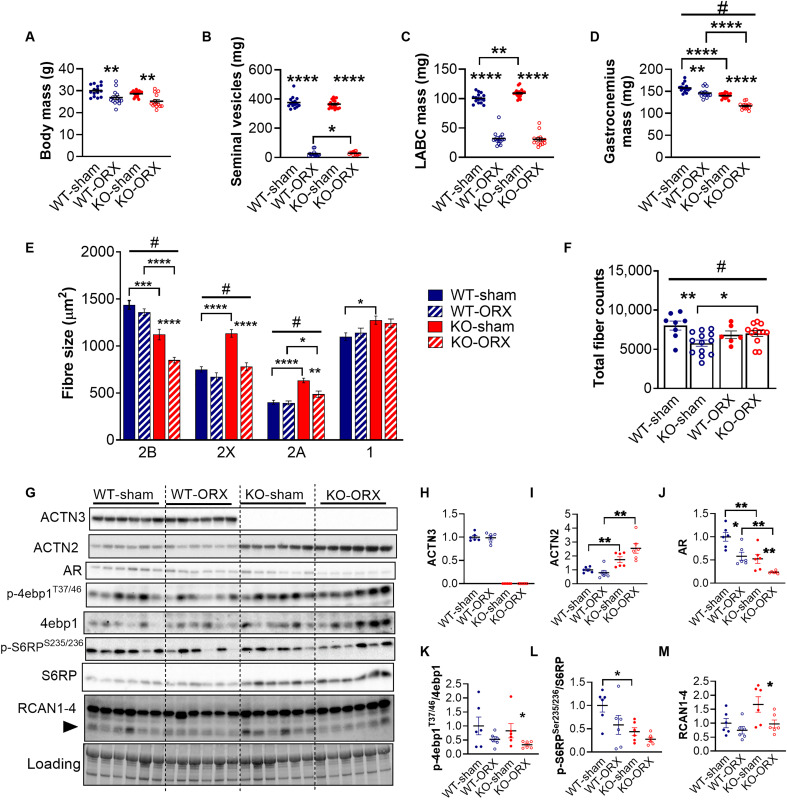
α-Actinin-3 deficiency differentially alters the muscle wasting and calcineurin signaling response induced by androgen deprivation. Orchidectomy (ORX) decreased (**A**) body mass, (**B**) seminal vesicle, (**C**) levator ani bulbocavernosus (LABC), and (**D**) gastrocnemius mass of WT and *Actn3* KO mice; KO showed greater atrophy for the gastrocnemius muscle compared to WT. (**E**) The size of fast 2B, 2X, and 2A fibers is reduced in KO-ORX, but not WT-ORX, gastrocnemius muscles compared to that in controls. (**F**) Muscles of orchidectomized WT, but not KO, show significant reductions in fiber count. (**G**) Western blot analyses quantify the effect of ORX in WT and KO gastrocnemius muscles on the expression of (**H**) ACTN3, (**I**) ACTN2, (**J**) AR, (**K**) p-4ebp1^Thr37/46^/4ebp1, (**L**) p-S6RP^Ser235/236^/S6RP, and (**M**) RCAN1-4. Data are represented as means ± SEM. *P < 0.05, **P < 0.01, ***P < 0.001, and ****P < 0.0001 by Mann-Whitney *U* test; #*P* < 0.05 by two-way ANOVA.

To determine whether baseline reductions in AR signaling with α-actinin-3 deficiency influence the skeletal muscle response to androgen deprivation, orchidectomy (ORX) was performed on adult male WT and *Actn3* KO mice (aged 8 to 10 weeks) and compared to sham-operated mice. After 12 weeks, ORX induced significant reductions in both WT-ORX and *Actn3* KO-ORX mice relative to those in sham controls in body mass, lean mass, BMD, bone mineral content (BMC), the mass of seminal vesicles, and the levator ani bulbocavernosus (LABC) muscle, as well as an increase in % fat mass, confirming complete androgen deprivation in both genotypes (Fig. 2, A to C, and table S2).

ORX also induced significant decreases in the mass of all hindlimb muscles examined in both WT-ORX and KO-ORX, except for the soleus; there is also no effect on heart mass (table S3). However, KO-ORX mice showed significantly greater atrophy for the gastrocnemius muscle compared to WT-ORX (WT-ORX, −7.3%; KO-ORX, −16.7%; *F*_1,59_ = 7.666, *P* = 0.0075) (Fig. 2D). Similar trends are observed in the quadriceps, TA, extensor digitorum longus, and spinalis, suggesting that α-actinin-3 deficiency increases muscle wasting induced by androgen deprivation (table S3).

### *Actn3* KO-ORX muscles show fiber atrophy and decreases in calcineurin activity

Analysis of fiber size and number in the gastrocnemius muscle indicates differential mechanisms mediating muscle atrophy in WT-ORX and KO-ORX (Fig. 2, E and F). Two-way ANOVA confirmed a significant interaction between the effects of *Actn3* genotype and ORX on the size of fast 2B (*F*_1,47_ = 5.239, *P* = 0.0266), 2X (*F*_1,42_ = 11.62, *P* = 0.0015), and 2A fibers (*F*_1,40_ = 6.561, *P* = 0.0143) with KO-ORX muscles showing significantly smaller fast 2B, 2X, and 2A fibers compared to KO-sham, while the size of fast and slow fibers is not altered in WT following ORX (Fig. 2E). In contrast, ORX significantly decreased total fiber number in WT-ORX, but not *Actn3* KO-ORX; two-way ANOVA showed significant interaction between *Actn3* genotype and effect of ORX (*F*_1,35_ = 6.687, *P* = 0.014) (Fig. 2F). There is no change in fiber-type proportions in either genotype (fig. S2).

Further assessment of both WT-ORX and KO-ORX gastrocnemius muscles showed significant reductions in AR expression in both genotypes compared to sham controls (Fig. 2, G and J). Evaluation of the downstream changes in mTORC1 activity, which regulates cell size, metabolism, and growth, showed similar reductions in the ratio of phosphorylated to total 4ebp1 and S6RP expression in both genotypes (Fig. 2, K and L). We also examined RCAN1-4 expression, a marker of calcineurin activity, because calcineurin mediates the androgen-induced hypertrophic response of myotubes (*5*) and is also significantly increased in α-actinin-3–deficient muscles at baseline and in response to immobilization and denervation and exercise training (*25*, *29*). RCAN1-4 levels are similar between WT-ORX and WT-sham but are significantly decreased in KO-ORX relative those in to KO-sham (Fig. 2M).

### α-Actinin-3 deficiency alters the transcriptional response to androgen deprivation

**Fig. 3.**
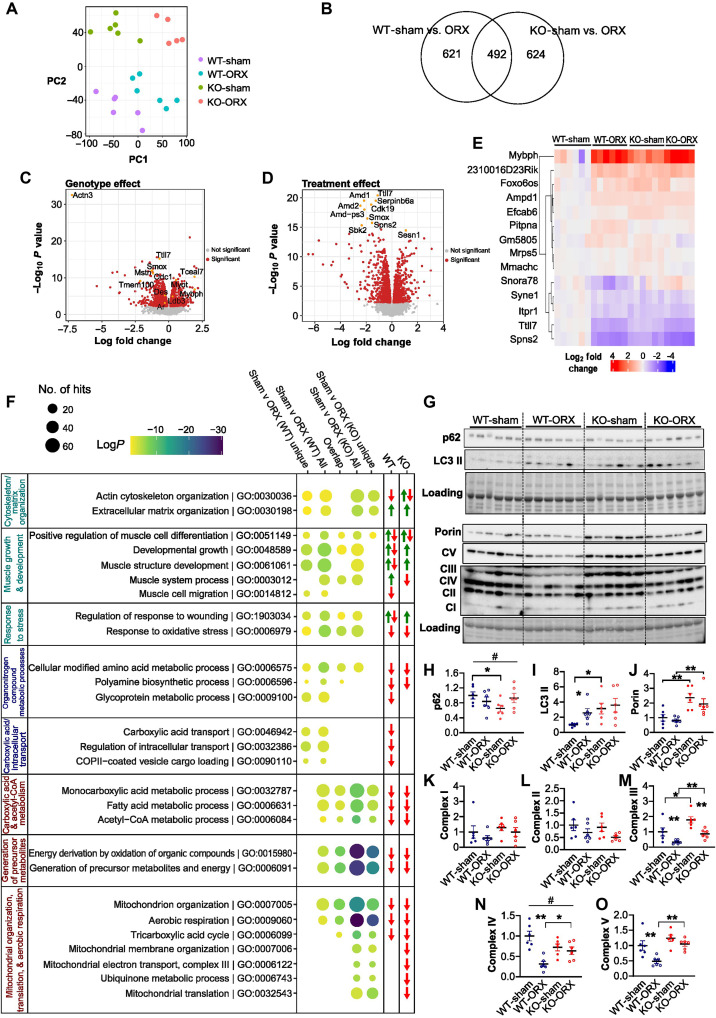
Transcriptomic analysis highlights the differential metabolic response to androgen deprivation with α-actinin-3 deficiency. (**A**) Principal components analysis (PCA) dot plot shows clear separation and grouping of samples by treatment (sham/ORX) on principal component 1 (PC1) and *Actn3* genotype (WT/KO) on PC2. (**B**) Venn diagram (*q* < 0.01) shows a comparable number of differentially expressed genes for WT and KO in response to ORX, along with 492 genes that overlap for their effect in both genotypes compared to ORX. (**C** and **D**) Volcano plots highlight the differentially expressed genes (*q* ≤ 0.01, in red) due to the effect of (C) *Actn3* genotype and (D) ORX. (**E**) Heatmap shows expression in log_2_ fold change of the 14 statistically significant genes (*q* < 0.05) from the interaction test performed. Fold changes are calculated against the mean of WT-sham expression samples. (**F**) Summarized gene set enrichment analysis groups GO terms by commonality and illustrates the difference in response to ORX between WT (WT unique, in blue) and *Actn3* KO (KO unique, in red) along with those that are present in both genotypes (in teal). The size of the circles represents the number of hits within the GO term and statistical significance in log(*P*) is represented by the color of the circle. Green and red arrows denote increased and decreased expression, respectively, of genes enriched in each GO term in ORX samples relative to that in sham. CoA, coenzyme A. (**G**) Western blot analysis confirms differential *Actn3* genotype effects on the response to ORX for (**H**) autophagy marker p62 and (**I**) LC3 lipidation, as well as changes for (**J**) porin and (**K** to **O**) mitochondrial complexes I to V. Data are represented as means ± SEM; *P < 0.05 and **P < 0.01 by Mann-Whitney *U* test; #*P* < 0.05 by two-way ANOVA.

We further performed whole transcriptomic profiling in the gastrocnemius muscle to examine the mechanisms underlying the differential muscle wasting response with α-actinin-3 deficiency following ORX (Fig. 3). Principle components analysis of the filtered and processed count data confirmed the experimental grouping of gene expression by *Actn3* genotype and sham/ORX treatment (Fig. 3A). Differential expression testing for effects of ORX identified 1113 genes in WT and 1116 genes in *Actn3* KO muscles that were differentially expressed (*q* ≤ 0.01) in the expression, with 492 of these genes common between *Actn3* genotypes (Fig. 3B).

Tests of genotype effect (Fig. 3C) confirm the transcript-level knockout of *Actn3* expression in *Actn3* KO relative to that in WT. As expected, we observe reduced expression of *Ar*, *Smox*, *Odc1*, *Tmem100*, and *Mstn* (*27*) and increased expression of *Ldb3*, *Myot* (*35*), and *Tceal7* with α-actinin-3 deficiency. Of note, *Mybph*, an androgen responsive gene (*6*, *36*, *37*), is also up-regulated with α-actinin-3 deficiency at baseline. Similarly, volcano plot of the treatment effect (Fig. 3D) verifies the down-regulation of the polyamine biosynthesis genes *Amd1* and *Smox* in response to ORX among the top 10 genes that are differentially regulated in skeletal muscle (*38*). Testing for effects of interaction between *Actn3* genotype and treatment (*q* < 0.05) identified 14 genes (Fig. 3E), with the pattern of expression for these genes in KO groups showing remarkable similarity to WT-ORX.

We performed gene set enrichment analysis testing to further characterize the overall response to ORX with respect to *Actn3* genotype. For each contrast, the top 15 most significant (by *q* value) gene ontology (GO) terms were used to generate GO plots to examine both common and unique response to ORX relative to sham in WT and KO muscles (fig. S3); this is summarized and organized in groups on the basis of commonality using QuickGO in Fig. 3F (*39*). Arrows denote directionality of gene expression changes in ORX samples relative to those in sham for genes enriched in each GO term, within each genotype. When examining the GO terms that are enriched in response to α-actinin-3 deficiency and ORX, we observe that there are several overlapping pathways related to cytoskeleton/matrix organization, muscle growth and development, response to stress, polyamine biosynthesis, and cellular modified amino acid metabolism. The WT response to ORX shows further unique enrichment for these pathways and demonstrates additional enrichment for GO terms relating to carboxylic acid/intracellular transport, which is not observed in the KO response. Both WT and *Actn3* KO muscles also show common responses for many GO terms related to the metabolism of carboxylic acid and acetyl–coenzyme A, generation of precursor metabolites, and mitochondrial metabolic processes, but *Actn3* KO demonstrates a greater amount of enrichment, as well as unique enrichment of other GO terms associated with mitochondrial activity. The expression of genes enriched in GO terms related to energy generation and metabolic processes is typically reduced in WT and KO in response to ORX.

To verify a subset of these results, we examined specific markers of autophagy and mitochondrial activity in both WT and KO muscles because they are associated with muscle atrophy induced by ORX (*40*), and autophagy is activated in response to oxidative stress and deficiencies in essential amino acids and adenosine 5′-triphosphate (ATP) (Fig. 3, G to O) (*41*). Contrary to our previous reports (*27*), *Actn3* KO-sham muscles show significantly higher lipidated LC3 (LC3-II), a standard marker for autophagosomes, and reduced p62 expression relative to WT-sham muscles, consistent with increased autophagic flux with α-actinin-3 deficiency at baseline. Our results also show a significant interaction between the effects of *Actn3* genotype and ORX on p62 expression (*F*_1,20_ = 4.457, *P* = 0.0475); WT-ORX shows reduced p62 relative to WT-sham, consistent with up-regulated autophagy in WT following ORX, while p62 levels showed a trend for increase in KO-ORX relative to that in sham control (Fig. 3H). A similar trend is observed for the expression of LC3-II (*F*_1,20_ = 0.9108, *P* = 0.3513), with WT-ORX showing significantly increased LC3-II expression relative to WT-sham (Fig. 3I), but not between KO-sham and KO-ORX.

Assessment of voltage-dependent anion channel (VDAC)/porin expression as a marker of mitochondrial number show no detectable difference in expression in either WT-ORX or KO-ORX relative to that in sham controls, although porin expression is consistently higher in KO compared to that in WT (Fig. 3J). There is, however, a significant interaction between the effect of *Actn3* genotype and ORX on the expression of complex IV (*F*_1,20_ = 10.74, *P* = 0.0038). Consistent with previous reports, complex IV expression is reduced with ORX in WT muscles (*40*). A similar trend is also observed for complex V (*F*_1,20_ = 2.310, *P* = 0.1442) (Fig. 3, K to O). Despite reduced transcript expression of genes related to aerobic respiration, protein expression of porin, complexes I, II, IV, and V are not different between KO-ORX and KO-sham. In sum, these results indicate that, in response to androgen deprivation, the absence of α-actinin-3 in skeletal muscle maintained the protein expression of mitochondrial complexes, while other pathways and processes that are typically activated in response to stress, such as autophagy, are suppressed.

### α-Actinin-3 deficiency reduces the muscle growth response to DHT at the onset of puberty

**Fig. 4.**
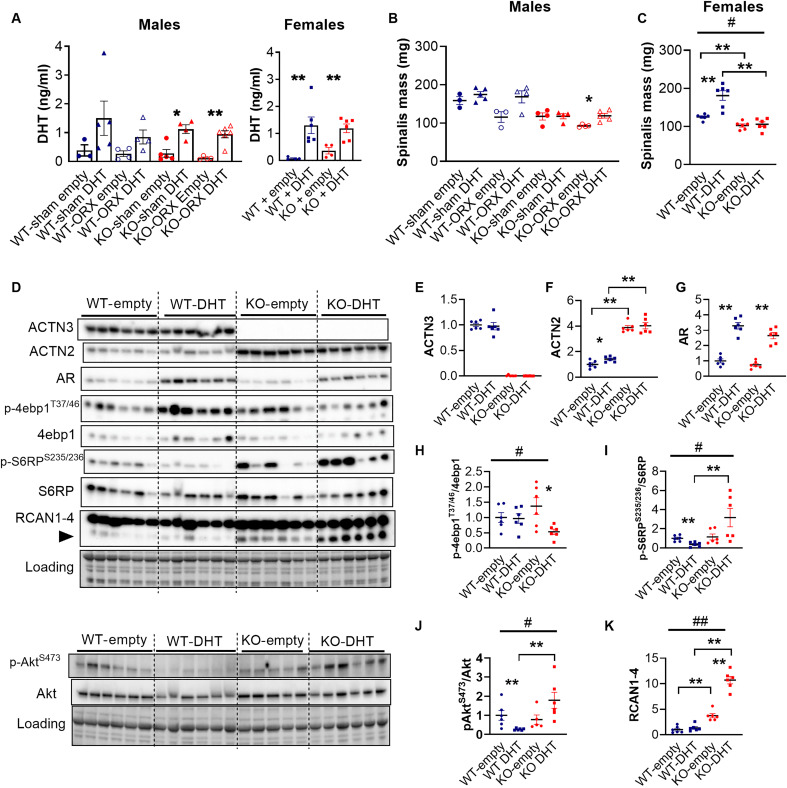
α-Actinin-3 deficiency decreases the muscle growth response to DHT at the onset of puberty. (**A**) Liquid chromatography–mass spectrometry (LC-MS) confirms increases in serum DHT in male (sham/ORX) and female WT and *Actn3* KO mice that were implanted with silastic tubing containing 10 mg of crystalline DHT for 6 weeks, relative to that in mice that were implanted with empty tubing. (**B**) DHT treatment prevented atrophy of spinalis muscles in orchidectomized WT and KO mice (**C**) Female WT-DHT, but not KO-DHT, mice show increases in spinalis mass relative to empty controls. (**D**) Western blot analysis quantifies the effect of DHT in female WT and KO spinalis muscles on the expression of (**E**) ACTN3, (**F**) ACTN2, (**G**) AR, (**H**) p-4ebp1^Thr37/46^/4ebp1, (**I**) p-S6RP^Ser235/236^/S6RP, (**J**) p-Akt^Ser473^/Akt, and (**K**) RCAN1-4. Data are represented as means ± SEM; *P < 0.05 and **P < 0.01 by Mann-Whitney *U* test; #*P* < 0.05 and ##*P* < 0.0001 by two-way ANOVA.

We further examined the effect of *Actn3* genotype on the response to androgen treatment. Silastic tubing that is either empty or filled with crystalline DHT is subcutaneously implanted at the onset of puberty (age 4 to 5 weeks) in intact (surgical sham) and orchidectomized male mice, as well as female WT and *Actn3* KO mice for 6 weeks. Liquid chromatography–mass spectrometry (LC-MS) analysis of sera analysis shows similar increases of DHT, 5α-androstane-3α,17β-diol (3-α-diol) and 5α-androstane-3β,17β-diol (3-β-diol) (the two diols are the primary metabolites of DHT) in all WT and *Actn3* KO mice implanted with DHT relative to those in respective controls (Fig. 4A and fig. S4).

DHT treatment prevented the loss in overall body weight and wet muscle mass with ORX in both WT-ORX and KO-ORX DHT mice compared to that in respective ORX mice implanted with empty controls and maintained seminal vesicle mass (table S4). The spinalis muscle, which is located adjacent to the site of implantation, shows the greatest hypertrophic response to DHT compared to other muscles. There is a trend for lower % increase in spinalis mass in DHT treated *Actn3* KO-ORX than WT-ORX (*F*_1,12_ = 1.656, *P* = 0.2225; WT-ORX DHT, +45.7%; *Actn3* KO-ORX DHT, +20.4%) (Fig. 4B). Similar results are observed in female mice and two-way ANOVA confirms a significant *Actn3* genotype effect on DHT response for overall body weight (*F*_1,20_ = 4.928, *P* = 0.0382), spinalis mass (*F*_1,20_ = 12.67, *P* = 0.0020), and heart mass (*F*_1,19_ = 9.791, *P* = 0.0055) (table S5). Relative to respective empty controls, female WT-DHT mice show greater increases than female KO-DHT mice for body mass (WT-DHT, +20.36%, *P* = 0.0022; KO-DHT, +5.0%, *P* = 0.4848), spinalis mass (WT-DHT, +45.2%, *P* = 0.0022; *Actn3* KO-DHT, +3.17%, *P* = 0.6991), and heart mass (WT-DHT, +25.8%, *P* = 0.0022; KO-DHT, +0.9%, *P* = 0.9307) (Fig. 4C and table S5). Similar trends are observed in the weight bearing muscles, quadriceps, and gastrocnemius (fig. S6A and table S5). This genotype difference in androgen response is specific to young mice at the onset of puberty, as repetition of this experiment in mature female WT and *Actn3* KO mice aged 12 weeks showed similar muscle hypertrophic response to DHT (fig. S5).

Fiber analyses in female gastrocnemius muscles showed that DHT treatment at the onset of puberty increased fast 2B and 2X fiber size in both WT and *Actn3* KO, but 2B fibers are consistently larger in WT muscles relative to those in *Actn3* KO (fig. S6B). Total fiber number for each fiber type is not different between any groups (fig. S6, C and D). Overall, these results suggest that α-actinin-3 deficiency significantly reduces the muscle growth response to DHT at the onset of puberty in female mice, with a similar trend observed in orchidectomized male mice. However, genotype differences in increases of wet muscle mass with DHT treatment cannot be explained by increases in fiber size.

### *Actn3* KO-DHT muscles show altered Akt/mTOR signaling and enhanced calcineurin activity

The expression of AR, α-actinins, and downstream muscle protein synthesis signaling is further assessed in female spinalis muscles from WT and *Actn3* KO mice that were treated with empty or DHT-filled implants at the onset of puberty (Fig. 4, D to Q). Following 6 weeks of DHT treatment, both WT-DHT and KO-DHT muscles show significant increases in AR expression compared to empty controls (Fig. 4G). α-Actinin-3 expression is unchanged with DHT in WT and KO (Fig. 4E), but WT-DHT shows a small significant increase in α-actinin-2 expression (Fig. 4F). Evaluation of 4ebp1 and S6RP activation (as markers of protein synthesis) and Akt shows significant interactions between the effect of *Actn3* genotype and DHT treatment on the ratio of p-4ebp1^Thr37/46^/4ebp1 (*F*_1,20_ = 4.860, *P* = 0.0394), p-S6RP^Ser235/236^/S6RP (*F*_1,20_ = 6.823, *P* = 0.0167), and p-Akt ^Ser473^/Akt (*F*_1,19_ = 9.724, *P* = 0.0057). Pair-wise comparisons with WT-empty show that WT-DHT muscles have significantly decreased p-S6RP^Ser235/236^/S6RP and p-Akt^Ser473^/Akt (Fig. 4, H to J). In contrast, KO-DHT shows significant decreases in p-4ebp1^Thr37/46^/4ebp1 and a trend for increased p-S6RP^Ser235/236^/S6RP relative to KO-empty.

We further examined changes in RCAN1-4 expression as a proxy for calcineurin activity. There is a highly significant differential *Actn3* genotype effect on RCAN1-4 in response to DHT (*F*_1,20_ = 51.97, *P* < 0.0001). RCAN1-4 expression is consistently higher in KO muscles compared to that in WT regardless of treatment, and, while DHT treatment did not alter RCAN1-4 levels in WT muscles, RCAN1-4 is significantly increased in KO-DHT muscles relative to that in KO-empty (Fig. 4K).

### α-Actinin-3 deficiency alters the transcriptional response to DHT in muscles of young female mice

**Fig. 5.**
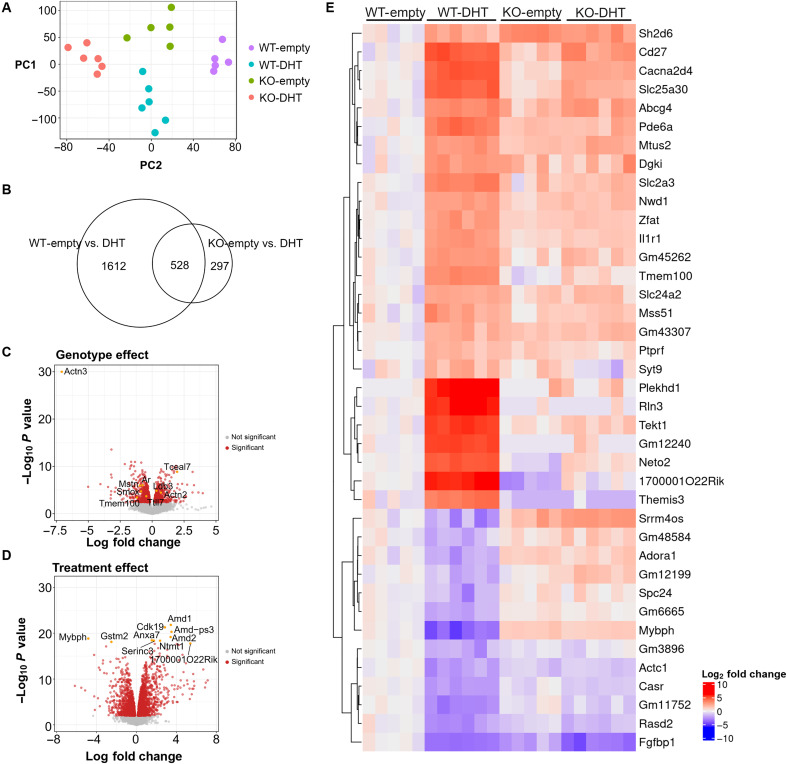
α-Actinin-3–deficient muscles show fewer differentially expressed genes in response to DHT. (**A**) PCA plot shows clear separation and grouping of samples by *Actn3* genotype (WT/KO) and treatment (empty/DHT) in two dimensions. (**B**) Venn diagrams (*q* < 0.01) illustrate greater differential gene expression for WT (1612 unique genes) in response to DHT compared to KO (297 unique genes), along with 528 genes that have an overlap of effect in both genotypes. (**C** and **D**) Volcano plots highlight the differentially expressed genes (*q* ≤ 0.01, in red) due to the effects of (C) *Actn3* genotype and (D) DHT. (**E**) Heatmap shows the log_2_ fold change of genes that are statistically significant from the interaction between *Actn3* genotype and DHT treatment (*q* ≤ 0.01, log_2_ fold change ≥ 2). Fold changes are calculated against the mean of WT-empty expression samples.

We further assessed global changes in the skeletal muscle transcriptome in spinalis muscles of female WT and *Actn3* KO mice treated with DHT at the onset of puberty. Principle components analysis demonstrates clear separation of samples based on DHT treatment and *Actn3* genotype (Fig. 5A). DHT treatment results in significant differential expression (*q* ≤ 0.01) of 2140 genes in WT and 825 genes in *Actn3* KO muscles, consistent with a reduced response to DHT with α-actinin-3 deficiency; of these, 528 genes are common to both WT and *Actn3* KO (Fig. 5B). Volcano plots of *Actn3* genotype (Fig. 5C) and effects of DHT (Fig. 5D) highlight many of the expected differentially expressed genes that are associated with these treatments (*37*).

**Fig. 6.**
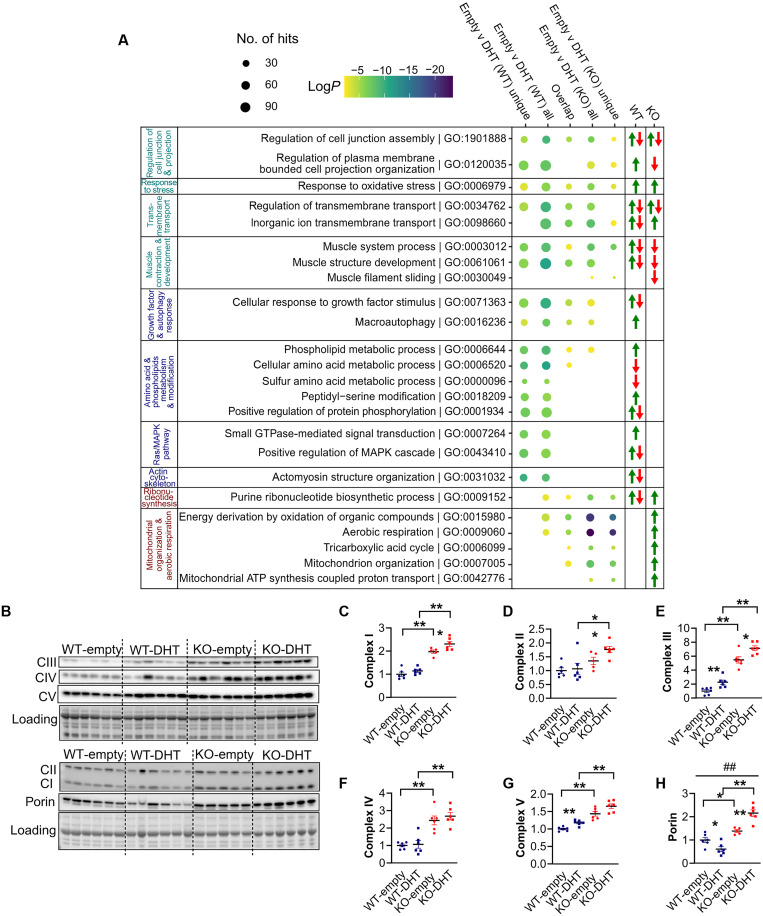
α-Actinin-3 deficiency alters the muscle transcriptomic response to DHT with preferential activation of mitochondrial metabolic processes. (**A**) Gene set enrichment analysis was performed to compare the difference in DHT response between WT (WT unique) and *Actn3* KO (KO unique) along with those representing commonality. A representative list of the top GO terms for each contrast is shown. Groups in teal are common in WT and KO, groups in blue denote GO terms with unique enrichment in WT, while groups in red show further unique enrichment in KO. Green and red arrows denote increased and decreased expression, respectively, of genes enriched in each GO term in DHT samples relative to that in empty. GTPase, guanosine triphosphatase. (**B**) Western blot analysis confirms differential *Actn3* genotype effects on the response to DHT for mitochondrial complexes I to V (**C** to **G**) and (**H**) porin. Data are represented as means ± SEM; *P < 0.05 and **P < 0.01 by Mann-Whitney *U* test; ##*P* < 0.0001 by two-way ANOVA.

Testing for effects of interaction between *Actn3* genotype and DHT treatment (*q* ≤ 0.05) identified 624 genes; heatmap of gene expression changes of the top genes (*q* ≤ 0.01, log_2_ fold change ≥ 2) shows minimal response to DHT in *Actn3* KO muscles (Fig. 5E). To determine how WT and *Actn3* KO mice differentially respond to DHT, a gene set enrichment analysis is performed to examine both commonly activated and uniquely activated pathways based on *Actn3* genotype. The top 15 most significant GO terms for each contrast are recorded (fig. S5); Fig. 6A shows a representative list organized into groups on the basis of QuickGO (*39*). Arrows denote directionality of gene expression changes in DHT samples relative to empty for genes enriched in each GO term, within each genotype. In response to DHT, both WT and KO show commonality as well as unique gene sets associated with the regulation of cell junction and projection, oxidative stress response, transmembrane transport, and muscle contraction and development. There is also an overlap between genotypes for processes relating to growth factor and autophagy response, amino acid and phospholipid metabolism, ribonucleotide synthesis, and aerobic respiration. However, only WT shows further enrichment for processes related to amino acid metabolism and modification, Ras/mitogen-activated protein kinase (MAPK) signaling, and actin cytoskeleton organization, while KO shows further unique enrichment of GO terms and increased expression of genes for ribonucleotide synthesis and mitochondrial organization and metabolism.

To confirm the differential activation of mitochondrial metabolism in response to DHT with α-actinin-3 deficiency, we further assessed the protein expression of mitochondrial complexes and VDAC/porin in WT and KO spinalis muscles (Fig. 6, B to H). KO muscles show higher expression of complexes I to V and porin compared to WT, regardless of treatment. In contrast to the effects of ORX, DHT treatment increased expression of complexes III and V in WT muscles relative to that in empty controls, while KO-DHT muscles showed significant increases for complexes I, II, and III relative to KO-empty. Testing for interaction effects between *Actn3* genotype and DHT treatment for expression of complexes I to V did not reach statistical significance, but there was a significant interaction effect on the expression of porin (*F*_1,19_ = 26.93, *P* < 0.0001), with WT-DHT muscles showing reductions in porin, while KO-DHT muscles show increased expression relative to controls. Together, these results are consistent with an increase in mitochondrial number and activity with α-actinin-3 deficiency in response to DHT.

### α-Actinin-3–dependent mediators of androgen response in skeletal muscle

**Fig. 7.**
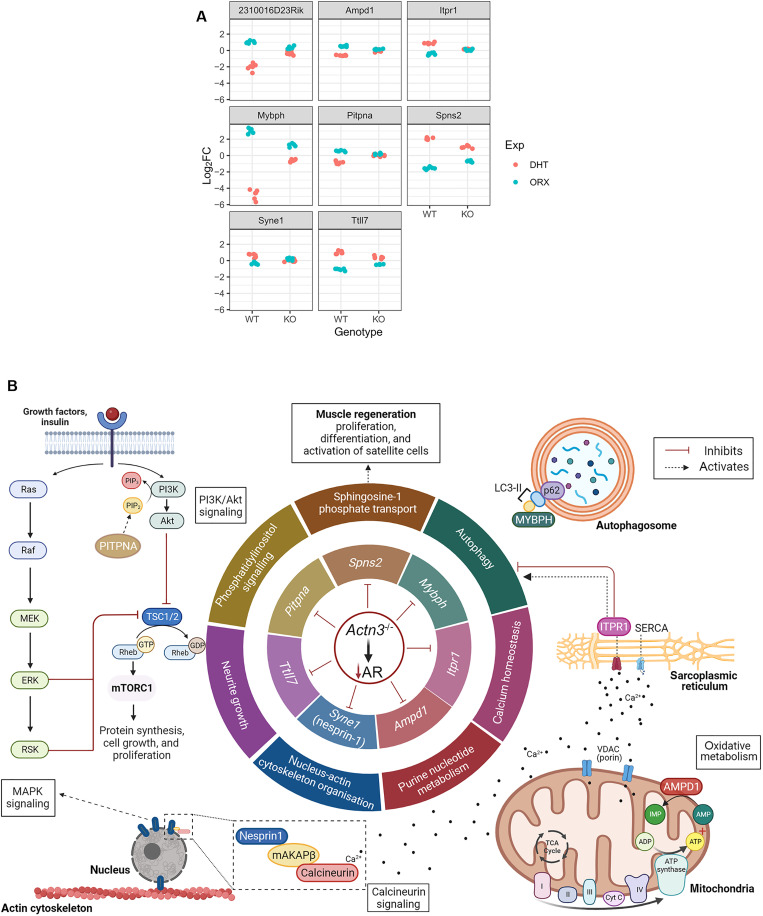
α-Actinin-3–dependent mediators of androgen response in skeletal muscle. (**A**) Log_2_ fold change (Log_2_FC) of differentially expressed genes (DEGs) that show significant interactions between ORX and *Actn3* genotype and also between DHT treatment and *Actn3* genotype. The DHT and ORX fold change responses for these DEGs are inverse. For both experiments, gene expression changes are lower in *Actn3* KO samples. (**B**) Schematic of proposed mechanistic changes with α-actinin-3 deficiency in response to androgen deprivation and DHT treatment. The absence of α-actinin-3 (which results in reduced muscle AR) inhibits/dampens the transcriptional response of *Mybph*, *Itpr1*, *Ampd1*, *Syne1*, *Ttll7*, *Pitpna*, and *Spns2* to changes in circulating androgens. The putative functions of these genes relate to autophagy, calcium homeostasis, purine nucleotide metabolism, nucleus-actin cytoskeleton organization, neurite growth, phosphatidylinositol signaling, and sphingosine-1 phosphate transport, respectively. The altered response of these genes to androgen deprivation and DHT treatment with α-actinin-3 deficiency may contribute to many of the differential downstream signaling [e.g., calcineurin, MAPK, phosphatidylinositol 3-kinase (PI3K)/Akt/mTORC1, autophagy, and oxidative metabolism] that influence muscle mass. The gene *2310016D23Rik* is also differentially expressed but is excluded from the schematic because its function is unknown. Created with BioRender.com. Seto, J. (2025) https://BioRender.com/0dv2hot.

To determine the specific mediators of androgen response that are ACTN3 dependent, we examined the differentially expressed genes that show significant interaction between *Actn3* genotype and androgen deprivation, as well as *Actn3* genotype and DHT. A total of eight genes are identified: *Mybph*, *2310016D23Rik*, *Ampd1*, *Pitpna*, *Syne1*, *Itpr1*, *Ttll7*, and *Spns2* (Fig. 7A)*.* The direction of change in expression of these genes relative to those in controls is inverse with androgen deprivation and DHT in both WT and KO samples, indicating that these genes are androgen responsive. However, the magnitude of fold changes is smaller in KO relative to that in WT, suggesting that the androgen-dependent regulation of these genes also requires the presence of α-actinin-3, despite there being no reports of direct or indirect interactions of these gene products with the α-actinins.

The putative functions of these genes vary greatly, ranging from autophagy (*Mybph*), calcium homeostasis (*Itpr1*), purine nucleotide metabolism (*Ampd1*), phosphatidylinositol signaling (*Pitpna*), neurite growth (*Ttll7*), sphinogosine-1 phosphate transport (*Spns2*), and nucleus-actin cytoskeleton organization (*Syne1*). With the exception of *Ttll7* and *2310016D23Rik*, these genes have also been associated with muscle atrophy and disease (*42*–*54*). The functions of these genes correlate with many of the pathways identified in Figs. 4F and 6A, suggesting that the dampened transcriptional response of these genes may have mediated or contributed to many of the differential signaling changes with α-actinin-3 deficiency in response to androgen deprivation and DHT treatment (Fig. 7B).

## DISCUSSION

In this study, we show that homozygous inheritance of a common null polymorphism in the *ACTN3* gene results in significantly reduced muscle AR protein expression in humans and mice, in both males and females. This finding is remarkable because muscle AR has been shown to be a key regulator of muscle force production, fatigue resistance, sarcomeric organization, and fiber-type distribution in various male ARKO models (*6*, *26*, *37*, *55*–*59*), and androgen sensitivity in skeletal muscle is strongly correlated with AR expression (*60*). Given that α-actinin-3 deficiency is common in the general population, our results suggest that healthy adult human muscle function is sustainable with ~70% reduction in functional AR in muscle, albeit at some cost to sprint and power performance.

The reduction of AR in the testis of *Actn3* KO mice also had no effect on baseline levels of serum testosterone, luteinizing hormone, and seminal vesicle mass, indicating no specific effects on testosterone or sperm production (*61*). Fertility in *Actn3* KO mice appears normal; however, testis size is marginally but significantly increased. This may be an adaptive response to maintain serum testosterone. In support of our findings in mice, a recent genome wide association study of 425,097 UK Biobank study participants (aged 40 to 69 years) did not identify *ACTN3* rs1815739 as a genetic determinant of testosterone levels and related sex hormone traits in men and women (*62*), contrasting the previous report of an association between increased serum testosterone with the *ACTN3* 577R allele in elite Russian athletes (*21*). Overall, these data suggest that the phenotypic effects of α-actinin-3 deficiency in skeletal muscle are a direct consequence of reductions in AR and not of changes in circulating testosterone in the general population.

Our findings indicate that the effect of α-actinin-3 deficiency is specific to AR and does not impact ER and TR signaling, despite α-actinin-2 (which is up-regulated with α-actinin-3 deficiency) being a coactivator for these nuclear receptors (*10*). We also show that α-actinin-3 is directly and positively associated with AR expression in mammalian skeletal muscle, indicating that α-actinin-3 plays a specialized role in the regulation of AR signaling that cannot be compensated by increased expression of α-actinin-2 (*12*), even though they share 80% amino acid sequence identity (*63*). These results complement earlier findings of reduced *Actn3* (but not *Actn2*) expression in testicular feminized mice that lack functional ARs in the testis (*22*) and in Sertoli cell–specific ARKO mice (*64*), confirming the presence of a specific and direct relationship between α-actinin-3 andAR signaling.

Consistent with our hypothesis, decreased baseline AR with α-actinin-3 deficiency alters the muscle adaptive response to changes in circulating androgens. Specifically, young female and castrated male *Actn3* KO mice showed smaller increases in muscle mass following DHT treatment at the onset of puberty. Muscle mass increases with DHT treatment are most evident in the spinalis muscle in females, possibly due to its proximity to the implant, but similar trends are observed in the quadriceps and gastrocnemius muscles. α-Actinin-3 deficiency enhanced the atrophic response to androgen deprivation in the gastrocnemius (a highly androgen-sensitive muscle), although the overall whole-body response to ORX was not different from controls. The differential response in muscle adaptation to changes in androgen levels with α-actinin-3 deficiency is accompanied by differences in the activation of downstream mTORC1 and calcineurin-NFAT (nuclear factor of activated T cell) signaling. Both WT and *Actn3* KO muscles show a similar trend for reduced phosphorylation of mTORC1 substrates 4ebp1 and S6RP with androgen deprivation, but there was a genotype difference in the phosphorylation of these substrates in response to DHT. RCAN1-4 expression (as a marker of calcineurin signaling) is unchanged in WT muscles in response to DHT treatment and androgen deprivation; however, in *Actn3* KO muscle, RCAN1-4 is significantly increased with DHT treatment and markedly reduced in response to androgen deprivation.

It is unclear the extent to which differences in mTORC1 and calcineurin activation account for the increased atrophic response to androgen deprivation and reduced growth response to DHT in *Actn3* KO mice. The involvement of these pathways in muscle atrophy/hypertrophy in response to androgen deprivation and testosterone treatment, respectively, is widely disputed, with recent studies suggesting that mTORC1 has a limited role in testosterone-induced muscle growth (*5*, *24*, *65*–*69*). The lack of change in RCAN1-4 expression in WT-ORX and WT-DHT muscles in our study is also consistent with others that do not support a role for calcineurin in muscle growth (*70*). However, our finding of inverse responses in RCAN1-4 expression with androgen deprivation and DHT treatment that is specific to *Actn3* KO muscles strongly suggests that calcineurin signaling is involved in muscle adaptation to changes in testosterone in the absence of α-actinin-3 and reduced AR. We have previously reported increases in calcineurin activity in muscles from adult *ACTN3* 577XX humans at baseline and from early muscle development in *Actn3* KO mice (*25*, *27*). Up-regulation of this pathway in *Actn3* KO mice also enhances the response to exercise training, modifies the muscle adaptive response to denervation and immobilization, and slows the progression of muscular dystrophy in *mdx* mice (*25*, *29*, *71*). These results further confirm calcineurin signaling as a critical pathway that is preferentially activated in the absence of α-actinin-3 during muscle remodeling.

Hypothesis-free transcriptomic analyses provided additional insights into the molecular pathways that are differentially altered with α-actinin-3 deficiency in response to changes in androgens. Heatmaps of the differentially expressed genes with *Actn3* genotype and ORX or DHT treatment clearly illustrate a lack of change in gene expression in *Actn3* KO compared to that in WT, confirming that androgen sensitivity is reduced in α-actinin-3–deficient muscles. Comparison of the expression profiles of the significant interaction genes in male KO-sham and WT-ORX muscles also show remarkable similarity, suggesting that α-actinin-3–deficient muscles in some respects are already in a “precastrated” state. Muscle AR expression levels of KO-sham are similar to that of WT-ORX, and this could underpin the lower body mass and lean mass seen with α-actinin-3 deficiency at baseline.

Results from our gene set enrichment analyses show that androgen deprivation in male mice and DHT treatment in female mice at early puberty commonly alter expression of genes involved in oxidative metabolism, amino acid catabolism, oxidative stress, muscle growth, and development, regardless of genotype. Myofiber AR expression has been shown to directly influence expression of these genes (*37*, *59*, *72*); hence, the coactivation/repression of these processes in response to changes in circulating androgens is consistent with published reports. ORX has been shown to reduce AR expression and downstream mTORC1 protein synthesis signaling as well as activate pathways that regulate autophagy and mitochondrial activity (*68*, *69*, *73*). In contrast, DHT increases cell proliferation, ATP production, amino acid transport, and protein synthesis through the epidermal growth factor receptor and MAPK activation (*74*, *75*).

Our finding that WT and KO show differential enrichment of these pathways in response to ORX and DHT suggest that transcription of many of these genes is also α-actinin-3 dependent, in addition to being sensitive to changes in androgens. For example, GO terms related to the MAPK cascade, amino acid metabolism, and modification are only enriched in WT-DHT muscles, suggesting that α-actinin-3 expression is necessary for activating transcription of these genes, consistent with the suppression of muscle growth in *Actn3* KO mice in response to DHT treatment. Similarly, processes related to carboxylic acid/intracellular transport and glycoprotein metabolism are only altered in WT-ORX. This is further supported by the differentially expressed genes that show significant interaction with *Actn3* genotype (Fig. 7) whose functions correspond to these processes. In sum, our data strongly suggest that α-actinin-3 is a key molecular partner in the coupling of these typical downstream signaling responses to changes in androgens.

Among these, myosin binding protein H (*Mybph*; MyBP-H) is of particular interest. MyBP-H is primarily expressed in fast-twitch muscle fibers and has a purported role in autophagy processes in cardiomyocytes as it colocalizes with LC3 at the autophagosome membrane (*42*, *43*). *Mybph* expression is higher in gastrocnemius muscles of male global ARKO mice (*24*) and is up-regulated in response to castration and down-regulated with testosterone/DHT treatment (*6*, *36*, *37*). *Mybph* is also consistently among the top genes up-regulated with α-actinin-3 deficiency at baseline in male (Fig. 3C) and female mice (*q* = 0.09, data S1). On this basis, persistent up-regulated *Mybph* could explain why muscle mass is reduced with α-actinin-3 deficiency. It also indicates that α-actinin-3 directly regulates *Mybph* transcription and thus may play an indirect role in autophagic activation.

Conversely, GO terms related to mitochondrial organization and activity are preferentially enriched with α-actinin-3 deficiency, both in response to ORX and DHT treatment. Evaluation of OXPHOS complexes shows reduction of complexes III, IV, and V in WT-ORX muscles, while expression levels of complexes IV and V are largely maintained in KO-ORX. In contrast, DHT treatment significantly increased porin expression in KO muscles but reduced porin levels in WT-DHT relative to those in controls. It is likely that mitochondrial remodeling could be a primary adaptive mechanism in skeletal muscles in the absence of α-actinin-3 and reduced AR, because *ACTN3* replacement (“rescue”) in *Actn3* KO muscles also specifically reduces oxidative metabolism, resulting in reduced force recovery after fatigue (*18*). Together, our findings indicate that the absence of α-actinin-3 results in a failure to coactivate many of the typical pathways in response to changes in androgens, leading to a reliance on leveraging mitochondrial remodeling, and calcineurin signaling to restore muscle homeostasis. Our results also highlight the pivotal role of α-actinin-3 in various processes associated with the regulation of protein turnover and muscle mass.

Last, it is important to note that our findings in the *Actn3* KO mouse are likely the aggregate effects of a global reduction in AR and not merely a consequence of reduced muscle AR. Data from GTEX indicate that the *ACTN3* 577XX genotype in humans is associated with a loss of *ACTN3* transcript expression in other tissues (fig. S8), suggesting that AR protein is likely reduced globally. We have shown that AR protein expression is reduced in skeletal muscles and testes of *Actn3* KO mice. This is an important distinction because muscle-specific ARKO mice do not display differential changes in hindlimb muscle mass following androgen deprivation or DHT treatment (*37*, *55*). Similarly, in our rAAV-mediated *ACTN3* gene rescue study (*18*), we did not observe muscle hypertrophy, and this was likely due to the increase of AR being muscle-specific. Others have demonstrated that AR levels in neurons may in fact play a greater role in regulating muscle mass (*76*). This raises the possibility that globally increasing α-actinin-3 expression (and thus AR) may increase muscle mass.

In conclusion, we have demonstrated that α-actinin-3 deficiency is associated with reduced AR protein expression in skeletal muscles and testes and influences androgen sensitivity in skeletal muscle. We have also shown that α-actinin-3 plays a distinct role in regulating the expression of genes in various pathways that influence muscle growth and metabolism. Our findings suggest that changes in mitochondrial activity are the primary adaptive response of α-actinin-3–deficient muscle to changes in androgen levels, consistent with the hypothesis that the *ACTN3* 577X allele was positively selected during recent human evolution due to enhanced muscle metabolic efficiency (*12*).

Furthermore, our results suggest that *ACTN3* R577X genotype will contribute to the clinical variability in patients with partial or mild androgen insensitivity syndrome and may also affect the health outcomes and treatment response of patients with prostate cancer who undergo ADTs (*77*). *Actn3* KO mice demonstrate increased muscle wasting following androgen deprivation, particularly in the weight-bearing gastrocnemius muscle, which is highly susceptible to atrophy in sarcopenic and cachexic conditions (*36*, *78*, *79*), suggesting that α-actinin-3–deficient patients receiving ADT may be at increased risk of muscle wasting. In addition, reduced muscle growth response to DHT in *Actn3* KO mice, specifically at the onset of puberty, indicates a specialized role for α-actinin-3 in androgen response during muscle development and maturation that warrants further investigation. Diminished androgen sensitivity during this critical phase of muscle growth could underlie the effects of α-actinin-3 deficiency on muscle mass and performance in elite athletes and the general population.

## MATERIALS AND METHODS

### Animals and ethics

All experiments were performed in male and female C57BL/6J mice. Mice were fed standard chow and water ad libitum and were maintained in a 12:12-hour cycle of light and dark at ambient room temperature (∼22°C). All experiments were approved by the Animal Ethics Committee of the Murdoch Children’s Research Institute (A917).

### Human muscle biopsies

Protein was extracted from human muscle biopsies obtained from female and male participants for analysis of AR expression. Female participants (*n* = 33) were healthy, premenopausal aged 18 to 37; *n* = 23 were not resistance trained and *n =* 10 were resistance trained (*80*). Male participants (*n* = 11) were also a combination of healthy sedentary and resistance trained subjects and were aged 20 to 40 (*81*). RNA was also obtained from vastus lateralis muscles of a separate cohort of moderately trained Caucasian men (aged 18 to 47; *n* = 24) and women (aged 21 to 45; *n* = 20) (*82*) for evaluation of *AR* gene expression by qPCR.

### Reverse transcription polymerase chain reaction

cDNA was generated using 5 ng of total RNA using iScript Reverse Transcriptase supermix (catalog no. 1708841) and C1000 Touch Thermal Cycler (Bio-Rad) as per manufacturer guidelines. RT-qPCR was performed in triplicate with the LightCycler 480 instrument (Roche Diagnostics) and LightCycler 384-well plates with sealing foil (Roche Diagnostics). Reaction volume of 10 μl contains 2× SensiFAST SYBR No-ROX mix (Bioline), 0.4 μl of each 10 μM forward and reverse primers, and 1 μl of cDNA. Amplification of the single polymerase chain reaction (PCR) product was confirmed using the melting point dissociation curve, and the crossing point (*Cp*) values were calculated using the LightCycler 480 software. For each gene, a standard curve was generated using serial dilution of plasmid DNA. Gene expression is then normalized to the geomean of two housekeepers *RPL27* and *RPL41*. Primers for reverse transcription–PCR reactions are as follows: *ACTN3* (forward: GACAGCTGCCAACAGGATCT and reverse: ATCCACTCCAGCAGCTCACT), *AR* (forward: CTTCGCCCCTGATCTGGTTT and reverse: CTCATTCGGACACACTGG-CT), *RPL27* (forward: GCAAGAAGAAGATCGCCAAG and reverse: TCCAAGGGGATAT-CCACAGA), and *RPL41* (forward: AAGTGGAGGAAGAAGCGAATG and reverse: TGGACC-TCTGCCTCATCTTT).

### ACTN3 over-expression studies

rAAV (recombinant adeno-associated viral) vectors containing a cytomegalovirus promoter (CMV) and full-length human *ACTN3* (rAAV-CMV-*ACTN3*) were delivered by intramuscular injection into the anterior compartment of the hindlimb of anesthetized Actn3 KO mice, as previously described (*18*). An injection containing 5 × 10^8^ to 5 × 10^10^ vector genomes (vg) of r-AAV-CMV-*ACTN3* in 30 μl of Hanks’ balanced salt solution was delivered throughout the length of the tibialis anterior (TA) muscle. The contralateral limb was given equivalent vector genomes of empty control vector. Mice were euthanized 6 weeks postinjection, and the TA muscles were harvested for analysis.

### Dual x-ray absorptiometry

Changes in body composition, including lean body mass, fat mass, bone mineral density (BMD), and bone mineral content (BMC), were assessed using GE Lunar Piximus2 dual x-ray absorptiometry scanner to assess mice 12 weeks post-ORX. All calculations were performed excluding the head and tail of the mice.

### Orchidectomy

Male mice aged between 8 and 10 weeks were given presurgical analgesia (buprenorphine 0.1 mg/kg) and anesthetized using isoflurane/oxygen. Incision was made via the abdomen to remove the fat pad, testes, and vas deferens; the vas deferens was sutured, and wound site was resealed with surgical clips for 10 days. Control animals received a sham orchiectomy, whereby the testes were surgically exposed but not cut and the wound site sealed with surgical clips. Animals were monitored and euthanized 12 weeks postsurgery.

### Implantation with DHT

Silastic tube implant prepacked with 10 mg of solid DHT or empty tubing (*83*) was subcutaneously implanted between the scapulae in male and female mice aged 4 to 5 weeks. Mice were given presurgical analgesia (buprenorphine, 0.1 mg/kg) and fully anesthetized using isoflurane/oxygen during implantation. Male mice were also orchiectomized at the same time to eliminate contributions from endogenous androgens. Mice were euthanized after 6 weeks of treatment.

### Steroid profile testing

The steroid profiles from sera including androgens, estrogens, testosterone (T), DHT, 5α-androstane-3α,17β-diol (3αDiol), 5α-androstane-3β,17β-diol (3βDiol), estradiol (E2), and estrone (E1) were assessed by LC-MS/MS assays (The ANZAC Research Institute) (*84*).

### Immunoblotting

Snap-frozen gastrocnemius or spinalis muscles were homogenized in 4% SDS lysis buffer and assessed for total protein concentration using Direct Detect Assay free cards (Merck Millipore, catalog no. DDAC00010-GR). Proteins were separated by SDS–polyacrylamide gel electrophoresis using stain-free precast midi-criterion gels (Bio-Rad) and then transferred to polyvinylidene fluoride membranes (Merck Millipore). These were blocked with 5% bovine serum albumin in 1× Tris-buffered saline with 0.1% Tween-20, probed overnight at 4°C with primary antibodies against α-actinin-3, α-actinin-2 (ab68204 and ab68167, Abcam), Akt1 [no. C73H10, Cell Signalling Technology (CST)], p-Akt (Ser^463^) (no. 4060, CST), mammalian target of rapamycin (mTOR; no. 2983, CST), p-mTOR (no. 5536, CST), p-4ebp1 (no. 2855, CST), 4ebp1 (no. 9452, CST), S6RP (no. 2217, CST), p-S6RP (no. 4856, CST), SQSTM1/p62 (no. 5114, CST), LC3B (no. 3868, CST), and p-AR (ab45089, Abcam). Blots were developed with Clarity Western ECL substrates (Bio-Rad) using Bio-Rad ChemiDoc. Densitometry was performed using Bio-Rad ImageLab software. Results were normalized to total protein and presented relative to those in WT control.

### Fiber morphometry analysis

Fiber typing was performed as previously described (*85*). Sections were imaged on the V-Slide Scanner (MetaSystems) and analyzed using Metamorph software (Molecular Devices).

### RNA-sequencing analysis

Total RNA was extracted from ~50 mg of mouse gastrocnemius (androgen deprivation experiment) or spinalis (DHT treatment experiment) by phenol chloroform extraction (1 ml of TRIsure solution; Bioline) and purified using the RNeasy Mini Kit (QIAGEN), and RNA integrity was determined using TapeStation (Agilent Technologies 2200). Libraries were prepared using the Illumina TruSeq Stranded Total RNA GOLD and sequenced on an Illumina NovaSeq 6000. Each sample was sequenced at 2 × 150 base pairs at ~40 M reads. Sample fastq files were processed using the RNASik pipeline (*86*). Sample reads were aligned using STAR (*87*), duplicates were marked using PICARD (*88*), and expressed reads were quantified using featureCounts (*89*). Differential gene expression analysis was performed in Degust (version 4.11) and R-4.3.2 limma (*90*). Lowly expressed genes were removed with a cutoff of 0.5 counts per million in at least two samples. *P* values were adjusted using the Benjamini-Hochberg method. As the experiment was structured as a pair of two factor experiments, genotype and treatment effects were probed separately and then compared by overlapping sets of differentially expressed genes. Interaction effects were modeled as a difference of differences. Gene set enrichment analysis was performed with Metascape (*91*) and grouped using QuickGO (*39*). To visualize data, figures were generated using R-4.3.2 ggplot2 and ComplexHeatmap (*92*). Directionality of GO terms was established by performing a second round of GO term enrichment of differentially expressed genes that were sorted by fold change into “up” and “down” sets and observing from which set the GO term is enriched.

### Statistics

Analyses were performed in GraphPad Prism (V7, GraphPad Software Inc.) and StataSE (StataCorp). As group sizes consisted of <12, two-sided, unpaired *t* tests using nonparametric statistics, Mann-Whitney *U* tests were applied using an α value of 0.05 for all analyses. Two-way ANOVA was applied to determine effects of genotype on interventions. Data were presented as individual points with the calculated group mean (line), or as bar graphs, with ±SEM error bars for each group.
