# Supplementary material for: ACTN3 genotype influences androgen response in developing murine skeletal muscle
Source: Sci Adv. 2025 Aug 27;11(35):eadw1059. doi: 10.1126/sciadv.adw1059 (PMC12383265; doi:10.1126/sciadv.adw1059)
Supplement: Supplementary file 1 — Figs. S1 to S8 Tables S1 to S5 Legend for data S1 [file sciadv.adw1059_sm.pdf]

Science Advances
AAAS

Supplementary Materials for

ACTN3 genotype influences androgen response in developing murine skeletal muscle

Kelly N. Roeszler *et al.*

Corresponding author: Jane T. Seto, jane.seto@mcri.edu.au

Sci. Adv. **11**, eadw1059 (2025)
DOI: 10.1126/sciadv.adw1059

The PDF file includes:

Figs. S1 to S8
Tables S1 to S5
Legend for data S1

Other Supplementary Material for this manuscript includes the following:

Data S1

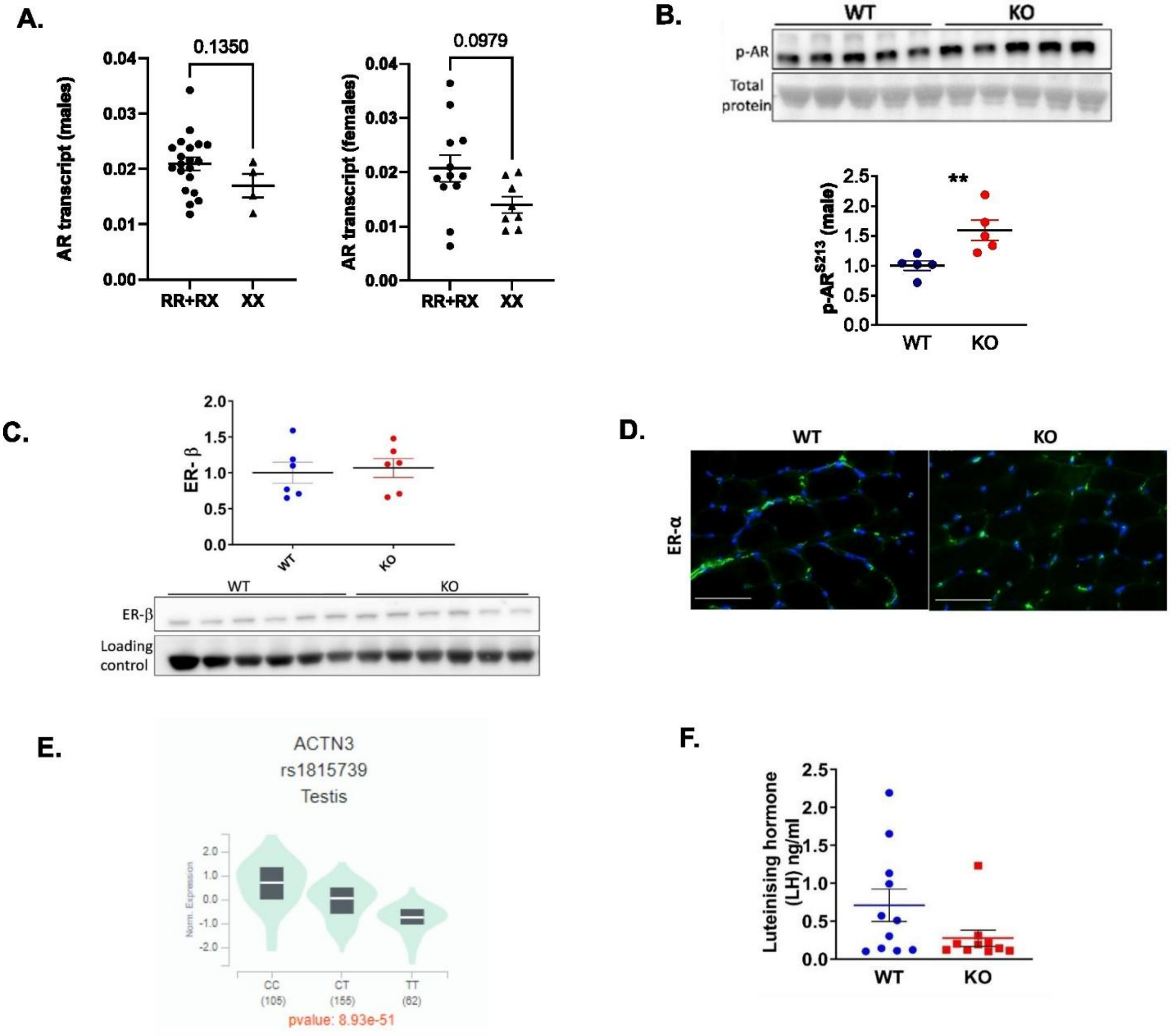

Fig. S1.

α-Actinin-3 deficiency alters androgen receptor expression in muscles from moderately trained people and mouse tissues, but not estrogen receptor and luteinising hormone levels in mice. A) qPCR analysis of *AR* in vastus lateralis muscles from a small cohort of moderately trained Caucasian men (aged 18-47; $N = 24$) and women (aged 21-45; $N = 20$) showed a trend for reduced *AR* expression in 577XX individuals compared to RR+RX. (B) p-AR^{Ser213} is upregulated in *Actn3* KO muscles compared to WT. (C) ER-β protein expression in female gastrocnemius muscle are similar between WT and *Actn3* KO. Expression levels are normalised to actin. (D) Immunostaining of ER-α shows similar staining intensities and localisation in female WT and *Actn3* KO muscles, scale bar = 50 μM. (E) eQTL scan in the GTEx testis dataset show that the R577X variant is significantly associated with *ACTN3* transcript expression $P = 8.93 \times 10–51$). (F) Circulating levels of luteinising hormone (LH) were determined by radioimmunoassay assay and are not significantly different between male WT ($N = 11$) and *Actn3* KO ($N = 10$) mice. Data are represented as mean ± SEM. $^{**}P < 0.01$ by Mann-Whitney U test.

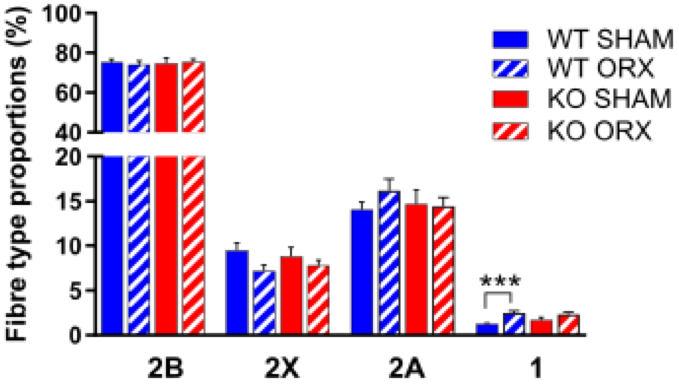

Fig. S2.

Fibre proportions in the gastrocnemius muscle of SHAM and ORX WT and *Actn3* KO mice. Fast fibre type proportions are not different in WT or *Actn3* KO muscles in response to castration, however slow type 1 proportions were marginally increased in WT. *N*=6-13 animals were analysed in each group. Data are represented as mean ± SEM. ****P* < 0.001 by Mann-Whitney U test.

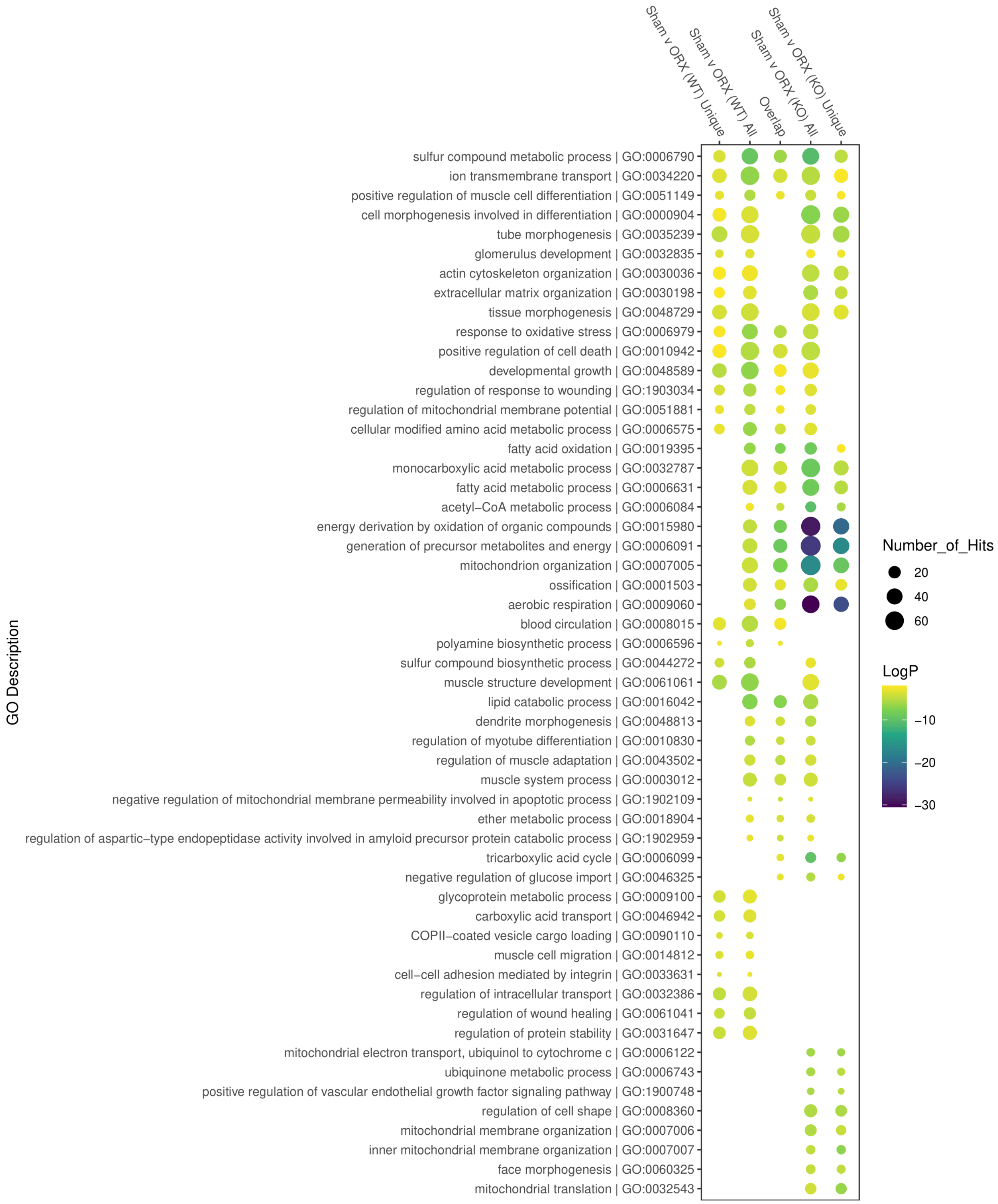

Fig. S3.

The top 15 gene ontology (GO) terms (by *q*-value) from each of the 5 contrasts demonstrate both common and unique response to orchidectomy relative to Sham in WT and KO muscle tissue samples.

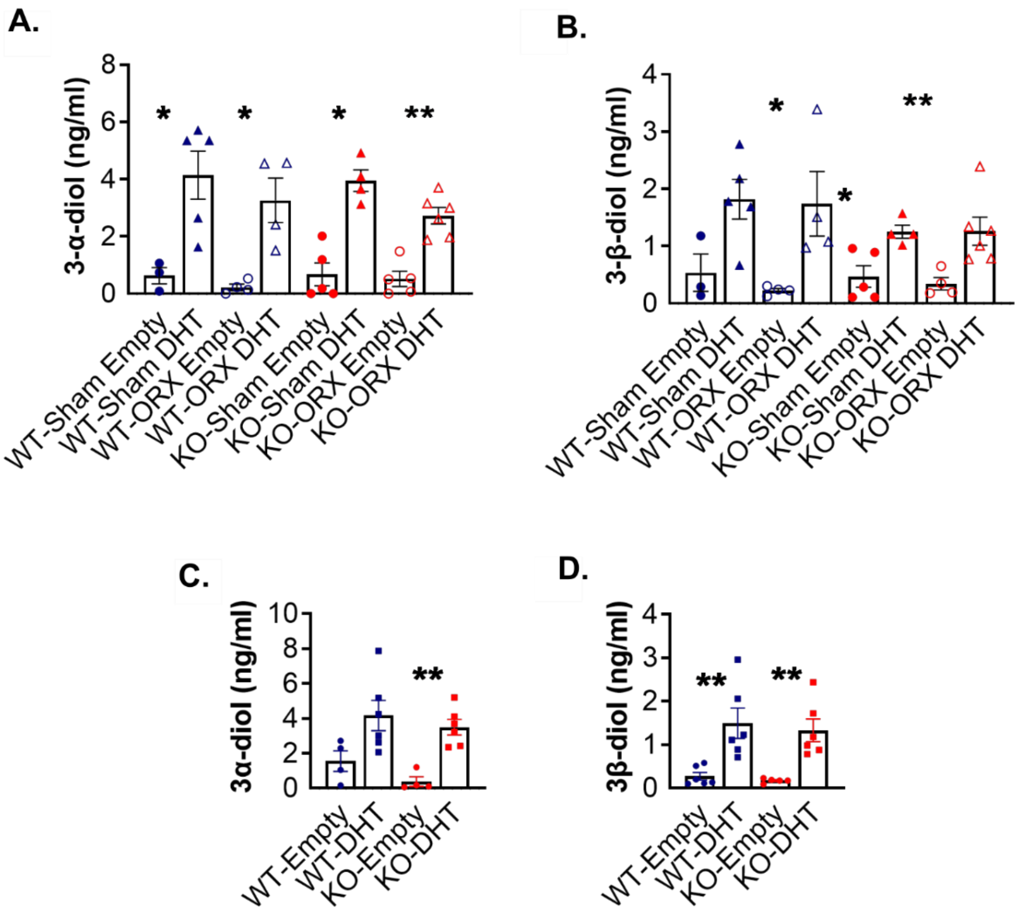

Fig. S4.

Liquid chromatography-mass spectrometry analysis of 3-α-diol and 3-β-diol levels (the primary DHT metabolites) in male (**A, B**) and female (**C, D**) mice. Data are represented as mean ± SEM; **P* < 0.05, ***P* < 0.01 by Mann-Whitney U test.

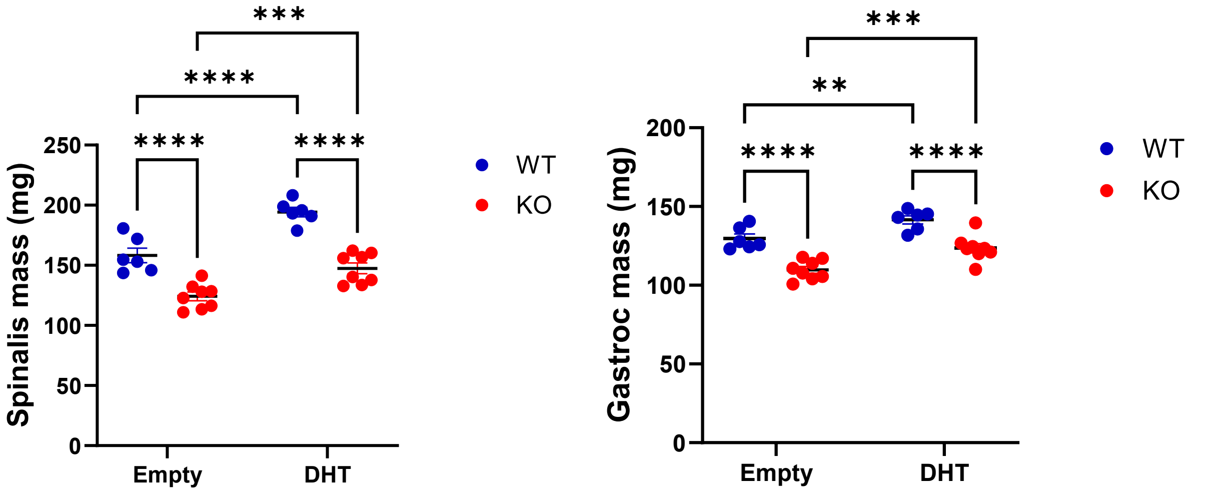

Fig. S5.
Mature 12 week old female WT and *Actn3* KO treated with Empty or DHT for 6 weeks. DHT treatment resulted in similar increases in mass of spinalis and gastrocnemius muscles relative to Empty in both WT and KO. There is no significant interaction effect between treatment and *Actn3* genotype for spinalis ($F(1,24)=1.964$, $P = 0.1739$) and for gastrocnemius ($F(1,24)=0.1327$, $P = 0.7188$).

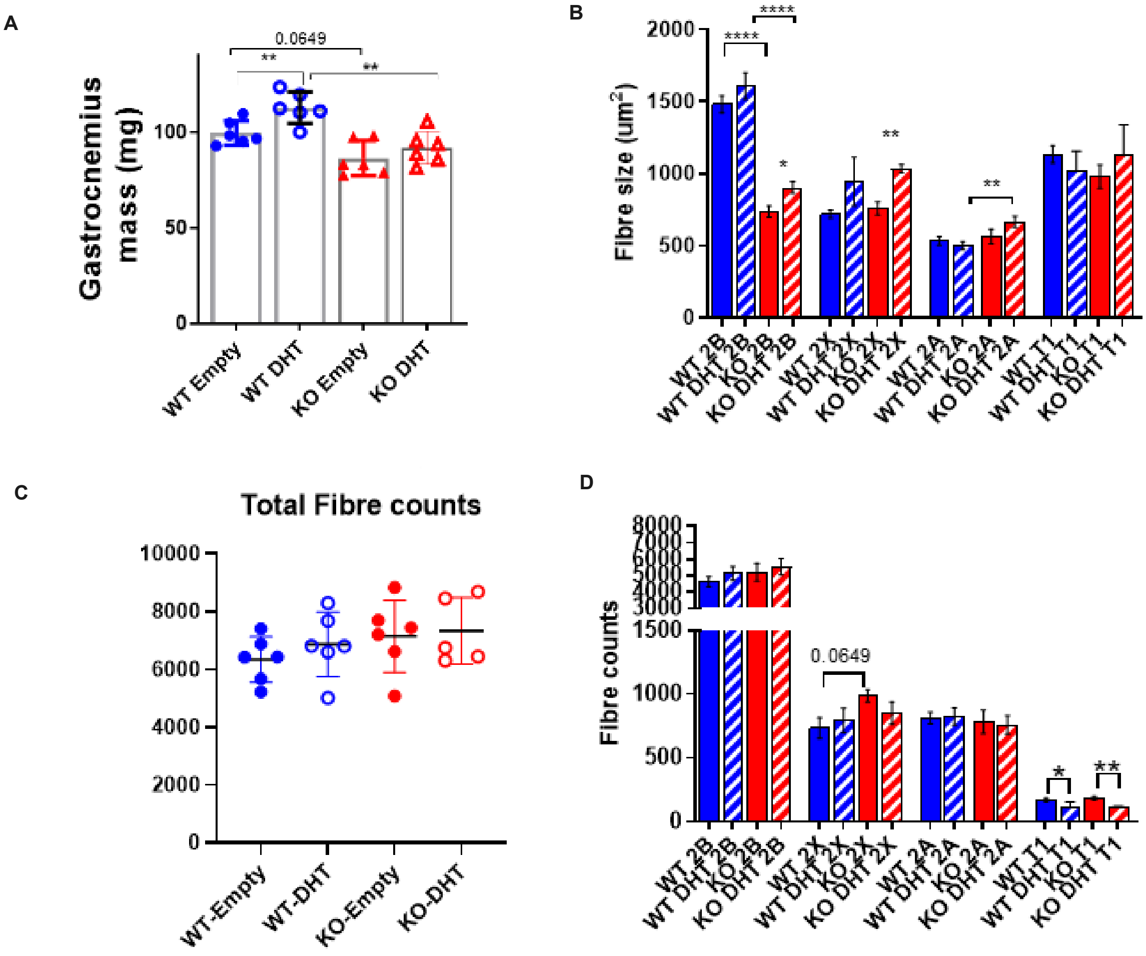

Fig. S6.

Morphological analysis of female WT and *Actn3* KO mouse gastrocnemius following Empty or DHT treatment. A) Gastrocnemius mass is significantly increased (13.3%) in female WT-DHT compared to WT-Empty, while in KO-DHT the increase in muscle mass is not significant relative to KO-empty (6.3%). B) DHT treatment increased fast 2B and 2X fibre size in both WT and *Actn3* KO, but 2B fibres are consistently larger in WT muscles relative to *Actn3* KO. C) Total fibre number and D) total fibre count for each fibre type is not different between any groups.

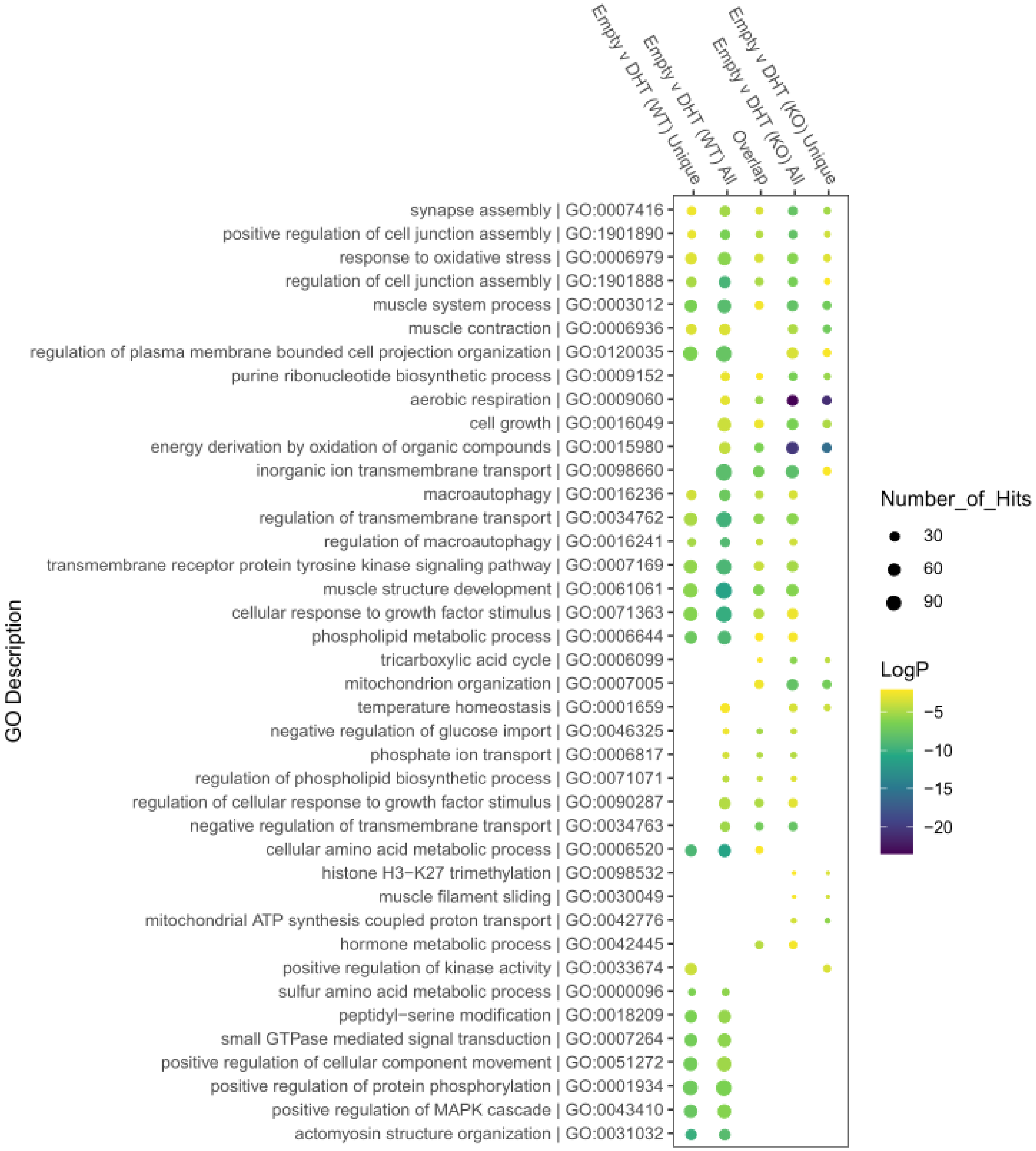

Fig. S7.

Dot plot of the top 15 most significant GO terms for each assessed contrast to investigate both common and unique responses to DHT relative to Empty in WT and KO muscles.

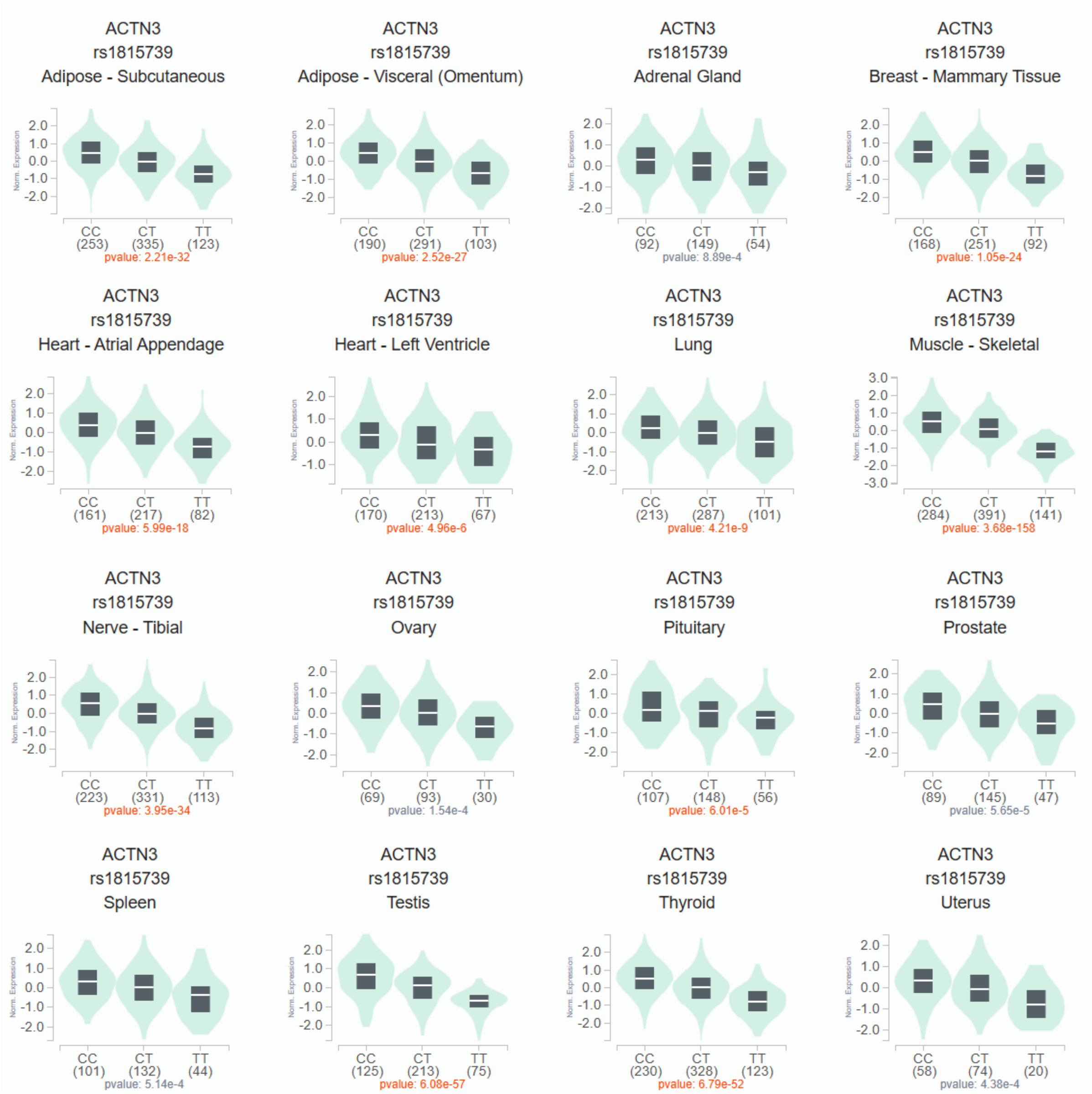

Fig S8.
eQTL scan in the GTEx dataset show that the R577X variant is significantly associated with *ACTN3* transcript expression in a number of other androgen sensitive tissues.

Table S1.

Baseline expression of genes associated with Androgen, Estrogen and Thyroid receptor signalling. *Ar, Smox, Odc1* are downregulated, but *Tceal7* is upregulated in *Actn3* KO relative to WT. In contrast, expression of genes associated with *Er* and *Tr* signalling are similar between WT and *Actn3* KO.

	**Gene**	**Gene name**	**Log fold change**	***P* value**	**Adj *P* value**
**Androgen signalling**	*Ar*	Androgen receptor	-0.29	0.0034	0.0252
	Smox	Spermine oxidase	-0.65	5.18E-06	1.99E-04
	Odc1	Ornithine decarboxylase 1	-0.41	0.001	0.0101
	Tceal7	Transcription elongation factor A like	2.32	5.81E-13	4.20E-10
**Estrogen signalling**	*Esr1*	Estrogen receptor α	0.09	0.4171	0.6165
	Esrra	Estrogen related receptor α	0.10	0.1449	0.3169
	Esrrb	Estrogen related receptor β	-0.04	0.6659	0.8073
	Esrrg	Estrogen related receptor γ	0.14	0.1415	0.3120
**Thyroid receptor signalling**	*Thra*	Thyroid hormone receptor α	0.07	0.1896	0.3765
	Thrb	Thyroid hormone receptor β	-0.02	0.8337	0.9145
	Thrsp	Thyroid hormone responsive	0.39	0.0621	0.1828

Table S2.

DXA analysis of WT and *Actn3* KO body composition following 12 weeks of androgen deprivation

	**WT**			***Actn3* KO**			**Two-way ANOVA**		
	SHAM (*N*=9)	ORX (*N*=9)	*P*-value	SHAM (*N*=10)	ORX (*N*=8)	*P*-value	Genotype	Treatment	Interaction
Lean mass (g)	23.28 ±2.17	20.03 ±2.20	0.0142	21.83±1.42†	18.56 ±1.16	<0.0001	0.017	<0.0001	0.9900
Fat mass (g)	6.07 ±1.03	6.47 ±1.10	0.9494	6.48 ±1.10	7.71 ±2.23	0.2263	0.1430	0.1500	0.4590
% Fat	20.67 ±2.95	23.93 ±4.19	0.1084	22.81 ±3.48	28.9 ±5.25	0.0166	0.012	0.0010	0.2970
BMD (g/cm^2)	0.053 ±0.0	0.049 ±0.0	0.0016	0.051 ±0.0*	0.047 ±0.0	<0.0001	0.0067	<0.0001	0.7940
BMC (g)	0.409 ±0.02	0.362 ±0.04	0.0194	0.401 ±0.03	0.358 ±0.02	0.0031	0.5380	<0.0001	0.8040

Table S3.
Effect of androgen deprivation on muscle mass and androgen responsive tissues.

	WT			*Actn3* KO			Two-way ANOVA		
	SHAM (*N*=15)	ORX (*N*=14)	*P*-value	SHAM (*N*=19)	ORX (*N*=15)	*P*-value	Genotype	Treatment	Interaction
**SV (mg)**	375.99±39.16	25.51±18.85	<0.0001	364.71±28.73	29.57±9.91	<0.0001	0.5967	<0.0001	0.2633
**LABC (mg)**	100.52±8.22	32.10±12.01	<0.0001	109.28±7.11†	30.62±10.77	<0.0001	0.1372	<0.0001	0.0363
**QUAD (mg)**	226.94±17.67	206.70±14.83	0.0037	193.25±10.7#	164.34±12.49	<0.0001	<0.0001	<0.0001	0.2252
**GST (mg)**	157.85±10.02	146.30±10.55	0.0059	140.1±5.76#	116.7±7.33	<0.0001	<0.0001	<0.0001	0.0075
**TA (mg)**	53.85±4.88	47.15±4.55	0.0008	50.47±3.13*	41.91±4.03	<0.0001	0.0002	<0.0001	0.3892
**EDL (mg)**	11.08±0.86	9.88±1.52	0.0066	10.53±0.65	8.86±1.15	<0.0001	0.0043	<0.0001	0.3729
**SOL (mg)**	9.39±1.19	8.25±1.36	0.0826	9.92±0.65	8.61±1.07	0.8977	0.0002	0.1347	0.1693
**SPN (mg)**	223.87±24.04	180.82±13.01	<0.0001	157.69±25.3#	121.93±17.21	<0.0001	<0.0001	<0.0001	0.5042
**Heart (mg)**	140.92±13.36	137.35±33.42	0.5556	153.32±29.09	124.21±19.71	0.2020	0.7612	0.8407	0.1134

Seminal vesicles (SV), levator ani bulbocavernosus (LABC), quadriceps (QUAD), gastrocnemius (GST), tibialis anterior (TA), extensor digitalis longus (EDL), soleus (SOL), spinalis (SPN) and heart (HRT). All samples were C57/BL6 male mice, had either SHAM or ORX surgery at aged 8-12 weeks. Data shown as mean ± SD. Mann-Whitney U pair-wise comparison tests.

Table S4.

Effect of *Actn3* genotype and DHT in male control and orchidectomised mice.

	**WT**						***Actn3* KO**						**Two-way ANOVA (ORX +/- DHT)**		
	SHAM + Empty (*N*=3)	SHAM +DHT (*N*=5)	*P*-value SHAM +/- DHT	ORX+ Empty (*N*=3)	ORX +DHT (*N*=4)	*P*-value ORX +/- DHT	SHAM + Empty (*N*=4)	SHAM +DHT (*N*=4)	*P*-value SHAM +/- DHT	ORX+ Empty (*N*=5)	ORX +DHT (*N*=5)	*P*-value ORX +/- DHT	Geno-type	Treat-ment	Inter-action
**BW (g)**	22.21 ±2.41	23.94 ±1.37	0.5714	19.58 ±0.21	25.40 ±3.08	0.0571	22.38 ±1.40	24.87 ±2.15	0.200	19.51 ±0.21	24.62 ±2.45	0.0079	0.6410	0.0002	0.8145
**SV (mg)**	237.23 ±2.90	353.10 ±0.37	0.0357	17.70 ±0.20	313.40 ±1.63	0.0571	232.28 ±0.56	365.30 ±0.50	0.0286	18.54 ±0.37	342.54 ±1.90	0.0286	0.4424	<0.0001	0.5695
**LABC (mg)**	71.43 ±2.05	92.28 ±1.59	0.0357	14.4 ±3.93[δ]	100.95 ±3.83	0.0571	87.6 ±21.07	96.08 ±6.43	0.4857	15.78 ±4.76	103.82 ±6.46	0.0159	0.4121	<0.0001	0.7711
**QUAD (mg)**	165.42 ±15.43	164.03 ±9.62	0.7857	147.58 ±12.70	179.23 ±21.39	0.1143	137.73 ±7.25	137.36 ±8.27	0.8857	123.16 ±6.59	140.89 ±12.66	0.8857	0.0005	0.0033	0.3310
**GST (mg)**	124.93 ±11.81	124.14 ±9.01	>0.9999	113.82 ±6.45	129.61 ±11.38	>0.9999	98.0 ±6.04	106.46 ±9.62	0.1143	90.79 ±4.70	108.84 ±2.11	0.1111	<0.0001	0.0002	0.7386
**TA (mg)**	41.95 ±5.39	42.11 ±2.63	>0.9999	35.05 ±0.95	42.88 ±4.22	0.0571	39.03 ±1.15	37.24 ±1.05	0.1143	33.66 ±1.03	40.31 ±3.42	0.0159	0.1867	0.0002	0.6855
**EDL (mg)**	9.38 ±2.41	8.53 ±1.37	0.7321	7.87 ±0.21	9.04 ±3.08	0.0571	8.58 ±1.40	8.54 ±2.12	0.6857	7.56 ±0.77	8.60 ±2.45	0.0635	0.2360	0.0525	0.8434
**SOL (mg)**	7.02 ±1.38	7.00 ±1.1	>0.9999	6.28 ±0.33	7.40 ±1.03	0.7000	8.56 ±0.91	8.28 ±0.49	0.6857	7.24 ±0.87	9.03 ±0.64	0.1111	0.0066	0.0031	0.4096
**SPN (mg)**	158.60 ±18.08	174.70 ±16.47	0.3929	115.60 ±24.08	168.45 ±31.45	0.2286	117.26 ±18.62	117.65 ±13.36	>0.9999	92.75 ±3.37	119.16 ±13.63	0.0159	0.0043	0.0023	0.2225
**HRT (mg)**	98.45 ±16.90	112.64 ±6.45	0.3810	96.00 ±4.76	121.88 ±4.01	0.0571	111.78 ±3.12	122.85 ±12.69	0.2286	92.92 ±3.66	117.76 ±13.07	0.0159	0.3806	<0.0001	0.8981

[δ]WT-Sham Empty *vs.* WT-ORX Empty LABC (*P*=0.0159). Body weight (BW), seminal vesicles (SV), levator ani bulbocavernosus (LA), quadriceps (QUAD), gastrocnemius (GST), tibialis anterior (TA), extensor digitalis longus (EDL), soleus (SOL), spinalis (SPN) and heart (HRT). All samples were C57/BL6 male mice, had either Sham or ORX surgery and received an empty or DHT aged 4-5 weeks at time of implant. 3-5 animals per genotype/treatment. Data shown as mean ± SD. Mann-Whitney U pair-wise comparison tests.

Table S5.
Effect of *Actn3* genotype and DHT on body, muscle and heart mass in females.

	WT			*Actn3* KO			Two-way ANOVA		
	Empty (*N*=6)	DHT (*N*=6)	*P*-value	Empty (*N*=6)	DHT (*N*=6)	*P*-value	Genotype	Treatment	Interaction
BW (g)	18.80 ±1.02	22.63 ±1.22	0.0022	19.51 ±1.47	20.48 ±2.29	0.4848	0.2759	0.0013	0.0382
QUAD (mg)	137. ±8.75	157.20 ±11.37	0.0087	124.60 ±12.65	127.70 ±13.52	0.8182	0.0003	0.0266	0.0962
GST (mg)	99.47±6.35	112.73±9.13	0.0087	86.32±9.13	91.73±8.49	0.3939	<0.0001	0.0105	0.2497
TA (mg)	33.85 ±1.66	41.42±2.83	0.0022	34.18±3.27	35.42 ±3.18	0.5887	0.0227	0.0912	0.9874
EDL (mg)	7.11 ±1.02	8.49 ±1.22	0.0130	7.28 ±1.47	7.58 ±2.29	0.5887	0.2781	0.0187	0.1152
SOL (mg)	5.98 ±0.52	7.03 ±0.89	0.0216	6.81 ±0.59	6.85 ±0.75	0.7316	0.2651	0.0718	0.0944
SPN (mg)	124.65 ±7.31	181.3 ±30.78	0.0022	102.5 ±10.28†	105.75 ±15.17	0.6991	<0.0001	0.0007	0.0020
HRT (mg)	89.03 ±5.90	112.02±5.60	0.0022	104.2±8.57†	105.13±12.03	0.9307	0.2543	0.0030	0.0055

†Baseline differences between WT and *Actn3* KO (Empty) ($P < 0.05$). Body weight (BW), quadriceps (QUAD), gastrocnemius (GST), tibialis anterior (TA), extensor digitalis longus (EDL), soleus (SOL), spinalis (SPN) and heart (HRT). All samples are from female C57/BL6 mice, aged 4-5 weeks at time of implant, 6 animals per genotype/treatment. Data shown as mean ± SD. Mann-Whitney U pair-wise comparison tests.

Data S1: Differentially expressed genes between control WT and KO (excel file)
